# Dual role of ferroptosis in embryonic development, cascade amplification regulatory mechanism and targeted intervention

**DOI:** 10.3389/fcell.2026.1912633

**Published:** 2026-09-02

**Authors:** Fan Meng, Shilong Li, Xue-Ting Wang, Nian Zhao, Dong Xiong, Weihang Zhang, Bin Liu, Shaoqiang Wang, Niannian Li

**Affiliations:** 1 Weifang People’s Hospital, Shandong Second Medical University, Weifang, Shandong, China; 2 School of Life Science and Technology, Shandong Second Medical University, Weifang, Shandong, China

**Keywords:** cascade amplification, developmental toxicity, embryonic development, embryonic development regulation, ferroptosis, targeted intervention

## Abstract

Ferroptosis is an iron-dependent regulated cell death driven by lipid peroxide accumulation. This review discusses its dual role in embryonic development. Physiologically, ferroptosis regulates cell fate and tissue remodeling through the synergistic GPX4-GSH and FSP1-CoQ10 pathways. Beyond passive cell death, iron-dependent trigger waves mediate spatially restricted cell clearance and limb muscle remodeling in avian models, integrating with Bone Morphogenetic Protein, Fibroblast Growth Factor, and Hedgehog signaling to sculpt tissue architecture as an integral component of normal morphogenesis. Pathologically, environmental stress and maternal genetic abnormalities activate ferroptosis, triggering an “iron overload → Fenton reaction → lipid peroxidation → GSH depletion/GPX4 inactivation” cascade that causes embryonic malformations. However, ferroptosis can also be directly induced by GPX4 inhibition or cysteine deprivation without prior iron overload. Current evidence relies mainly on animal models, and extrapolation to humans requires caution due to interspecies differences. Intervention targets include ferroportin regulation, GPX4/FSP1 activation, and NRF2 stabilization. Iron chelators, Ferrostatin-1, and antioxidants (NAC, astaxanthin) block the cascade at multiple nodes. Clinical use during pregnancy demands careful evaluation of off-target effects on physiological tissue sculpting. Future research should develop stage-specific and tissue-specific modulators. This review highlights the double-edged sword nature of ferroptosis, summarizes its regulatory network and intervention strategies, and proposes molecular markers (MDA, 4-HNE) for perinatal screening. Prospects include CRISPR/Cas9 and mesenchymal stem cell therapy, while multi-omics and human-specific models will accelerate clinical translation for preventing birth defects.

## 1 Introduction

**FIGURE 1:**
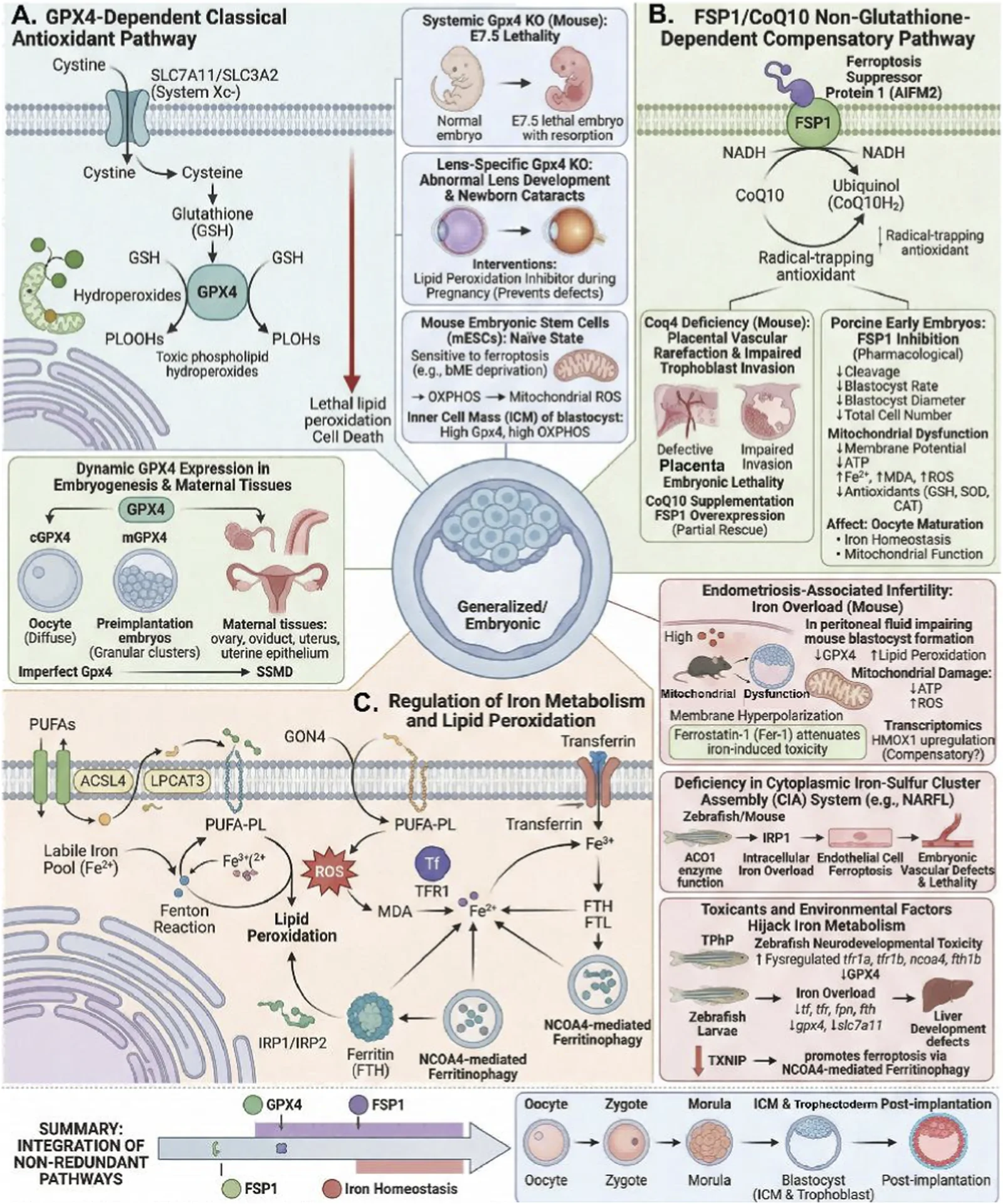
The three core regulatory pathways of ferroptosis and their roles in early embryonic development. **(A)** GPX4-dependent classical antioxidant pathway. Cystine enters the cell. The importer is a heterodimer. It consists of SLC7A11 and SLC3A2. Together they form System Xc^−^. Cystine is reduced to cysteine inside. Cysteine fuels GSH synthesis. GSH acts as a cofactor for GPX4. GPX4 reduces toxic phospholipid hydroperoxides (PLOOHs). PLOOHs come from lipid peroxidation. GPX4 converts them into harmless alcohols (PLOHs). This blocks lethal lipid peroxidation. GPX4 has different forms. Cytosolic GPX4 (cGPX4) works in the cytoplasm. Mitochondrial GPX4 (mGPX4) protects mitochondria. Both are needed for membrane integrity. GPX4 expression changes across embryogenesis. It also varies in maternal tissues. These include ovary, oviduct, uterus, and uterine epithelium. Somatic cell mitochondrial dysfunction (SSMD) reflects these patterns. In oocytes, cGPX4 is diffuse. In preimplantation embryos, mGPX4 forms granular clusters. Systemic Gpx4 knockout in mice causes lethality. This happens at E7.5. The embryo is resorbed. Lens-specific deletion is different. It causes abnormal lens development. Newborn cataracts follow. Lipid peroxidation inhibitors can prevent this. They are given during pregnancy. Naïve mouse embryonic stem cells (mESCs) have low GPX4. They rely on oxidative phosphorylation (OXPHOS). Mitochondrial ROS is high. These cells are sensitive to ferroptosis. bME deprivation triggers it. The inner cell mass (ICM) of blastocysts is different. It maintains high Gpx4. OXPHOS is also high. This ensures survival. **(B)** FSP1/CoQ10 non-glutathione-dependent compensatory pathway. FSP1 is the key protein here. It is also called AIFM2. It uses NAD(P)H as a cofactor. It reduces CoQ10 to ubiquinol. Ubiquinol is CoQ10H_2_. It traps lipid radicals. It blocks lipid peroxidation at the membrane. This works independently of GSH. CoQ10 synthesis needs enzymes. Coq4 is a key one. Coq4 deficiency in mice causes problems. Placental vascular rarefaction occurs. Trophoblast invasion is impaired. Embryonic lethality follows. CoQ10 supplementation helps. FSP1 overexpression also helps. Both provide partial rescue. In porcine early embryos, FSP1 inhibition matters. It is done pharmacologically. Cleavage rate drops. Blastocyst formation is reduced. Blastocyst diameter decreases. Total cell number falls. Mitochondrial dysfunction appears. Membrane potential drops. Fe^2+^ and ROS rise. Antioxidants deplete. Endometriosis is another context. It is associated with infertility. Iron overload accumulates in peritoneal fluid. Mouse blastocyst formation is impaired. GPX4 goes up. Lipid peroxidation increases. Heme oxygenase 1 (HMOX1) is upregulated. This may be compensatory. Ferrostatin-1 (Fer-1) can attenuate this. It reduces iron-induced toxicity. **(C)** Regulation of iron metabolism and lipid peroxidation. PUFAs are the starting material. ACSL4 and LPCAT3 handle them. ACSL4 is acyl-CoA synthetase long-chain family member 4. LPCAT3 is lysophosphatidylcholine acyltransferase 3. They esterify PUFAs into phospholipids. The products are PUFA-containing phospholipids (PUFA-PLs). These are prone to peroxidation. GON4 (germ cell-less 4, also known as NHLRC2) is a transcriptional regulator implicated in lipid metabolism and oxidative stress responses; its precise functional role in this circuit remains to be fully elucidated. Free ferrous ions (Fe^2+^) come from the labile iron pool (LIP). They drive the Fenton reaction. Hydroxyl radicals form. ROS also forms. Lipid peroxidation starts. Malondialdehyde (MDA) is one product. It is toxic. Iron enters cells via transferrin (TF). Ferric iron (Fe^3+^) binds TF. Transferrin receptor (TFRC) mediates uptake. Iron is stored in ferritin. Ferritin has two chains. Ferritin heavy chain (FTH) and ferritin light chain (FTL) form the complex. Iron can be released. NCOA4 mediates this. The process is ferritinophagy. Iron regulatory protein 1 (IRP1) and iron regulatory protein 2 (IRP2) control iron gene expression. They work post-transcriptionally. IRP1 has a dual role. With iron-sulfur clusters it is cytoplasmic aconitase 1 (ACO1). Cluster assembly needs the CIA system. NARFL participates. NARFL is nuclear prelamin A recognition factor-like. CIA deficiency causes problems. Intracellular iron overload occurs. Endothelial cell ferroptosis follows. Embryonic vascular defects result. Environmental toxicants also matter. Triphenyl phosphate (TPhP) is one example. It hijacks iron metabolism in zebrafish. Several genes are dysregulated. These include tfba, tfr1b, ncoa4, fth1b, ltf, fth, and fthl. Neurodevelopmental toxicity follows. In zebrafish larvae, iron overload occurs. ltf, fth, fthl go down. lgp4a and slc7a11 go up. TXNIP is thioredoxin interacting protein. It goes up. It promotes ferroptosis. It does so via NCOA4-mediated ferritinophagy. Liver developmental defects result. Summary. GPX4, FSP1, and iron homeostasis are three pathways. They are non-redundant. They integrate dynamically. This happens across embryonic development. The stages include oocyte, zygote, morula, blastocyst, and post-implantation embryo. The blastocyst has ICM and trophectoderm.

Regulatory cell death plays a crucial role in tissue remodeling and embryonic development. Among them, ferroptosis is an iron-dependent regulatory cell death process characterized by iron metabolism disorder and lipid peroxidation accumulation. It exhibits dual physiological and pathological effects during embryonic development, and its cascade amplification effect is particularly significant under pathological conditions. Ferroptosis is a positive feedback chain reaction initiated by iron overload, driven by lipid peroxidation, and mediated by the inactivation of the glutathione peroxidase 4 (GPX4)-glutathione (GSH) pathway (Figure 1A). This pathway depends on cystine import through the SLC7A11/SLC3A2 heterodimer, also called System Xc^−^. Cystine is reduced to cysteine inside the cell. Cysteine is the rate-limiting substrate for GSH synthesis. GPX4 uses GSH to reduce toxic phospholipid hydroperoxides (PLOOHs). PLOOHs are the direct executors of membrane damage. GPX4 has two main forms. Cytosolic GPX4 (cGPX4) works in the cytoplasm. Mitochondrial GPX4 (mGPX4) protects the mitochondrial membrane. Both are indispensable for embryonic cell survival. Systemic Gpx4 knockout in mice causes lethality at E7.5. Lens-specific deletion causes abnormal lens development and cataracts. Naïve mESCs with low GPX4 and high OXPHOS are sensitive to ferroptosis. The ICM of blastocysts maintains high GPX4 for protection. Dynamic GPX4 expression is seen across embryogenesis and maternal tissues. These include ovary, oviduct, uterus, and uterine epithelium. Somatic cell mitochondrial dysfunction (SSMD) reflects these expression patterns. Specifically, when intracellular free ferrous ions are overloaded, the Fenton reaction catalyzes the generation of a large number of hydroxyl radicals (·OH), which directly attack polyunsaturated fatty acids (PUFAs) on the cell membrane, initiating initial lipid peroxidation. The generated lipid peroxides (LPO) not only directly damage cell membrane integrity but also continuously consume GSH, leading to the depletion of the core reducing agent GPX4 and an irreversible decrease in enzyme activity. After GPX4 is inactivated, the unremovable LPO accumulates in large quantities intracellularly, which in turn further exacerbates the redox cycle of iron ions, forming a closed-loop cascade amplification loop of iron overload → lipid peroxidation initiation → GSH depletion/GPX4 inactivation → uncontrolled lipid peroxidation → further exacerbation of iron overload (Figures 1B,C). This loop is not the only mechanism. The FSP1-CoQ10 pathway provides backup defense. FSP1 is also known as AIFM2. It reduces CoQ10 to ubiquinol (CoQ10H_2_) using NAD(P)H. Ubiquinol traps lipid radicals at the membrane. Coq4 is required for CoQ10 synthesis. Coq4 deficiency causes placental vascular defects. In porcine embryos, FSP1 inhibition reduces blastocyst quality. Endometriosis involves iron overload andheme oxygenase 1 (HMOX1) upregulation. Ferrostatin-1 can attenuate this toxicity. Iron metabolism itself is tightly regulated. Transferrin (TF) binds ferric iron. TFRC mediates cellular uptake. FTH and FTL form the iron storage complex. NCOA4 mediates ferritinophagy for iron release. Iron regulatory protein 1 (IRP1) and iron regulatory protein 2 (IRP2) control iron gene expression post-transcriptionally. IRP1 doubles as cytoplasmic aconitase 1 (ACO1). The CIA system with NARFL (nuclear prelamin A recognition factor-like, also known as CIAPIN1) assembles its iron-sulfur cluster. PUFA-PL substrates are generated by ACSL4 and LPCAT3. GON4 (germ cell-less 4, also known as NHLRC2) is a transcriptional regulator implicated in lipid metabolism and oxidative stress responses; however, its precise functional role in the ferroptosis regulatory circuit remains to be fully elucidated. Environmental toxicants like TPhP dysregulate iron genes in zebrafish. These include tfba, tfr1b, ncoa4, fth1b, ltf, fth, and fthl. Thioredoxin-interacting protein (TXNIP) promotes ferroptosis through NCOA4-mediated ferritinophagy. Ultimately, this triggers large-scale feroptosis in a short period of time, causing irreversible damage to embryonic development. As a crucial part of the programmed regulation of embryonic development, this article reviews the cascade amplification mechanism, dual role, and targeted intervention of ferroptosis in embryonic development, providing new insights for a deeper understanding of embryonic development mechanisms and theprevention and treatment of birth defects.

## 2 Molecular mechanisms of ferroptosis and their expression and function in early embryonic development

### 2.1 GPX4-GSH-dependent glutathione classical antioxidant pathway

**FIGURE 2:**
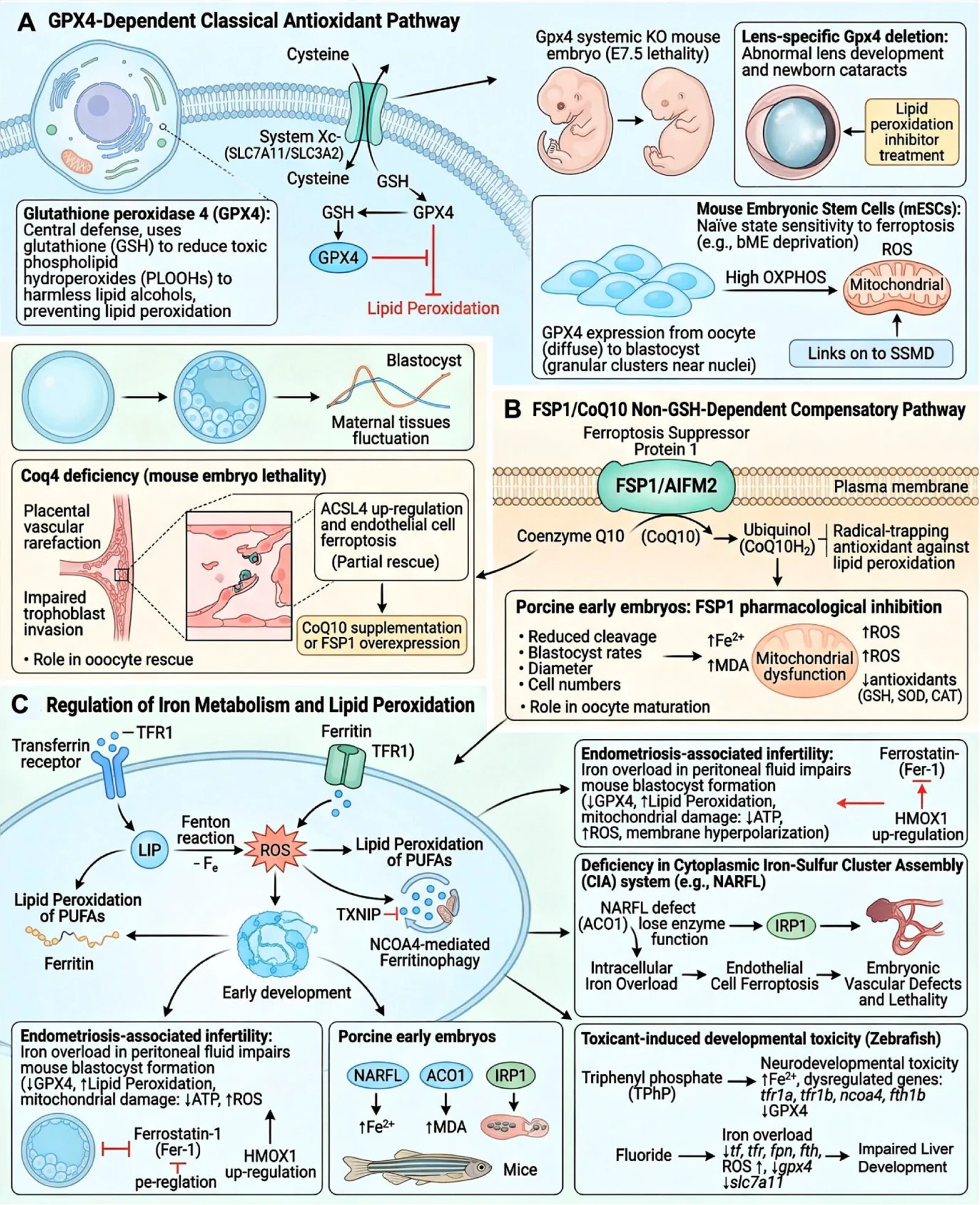
Core functions and evidence integration of ferroptosis regulatory pathways in early embryonic development. **(A)** Glutathione peroxidase 4 (GPX4)-dependent classical antioxidant pathway: As the core inhibitory axis of ferroptosis, it maintains embryonic cell survival by clearing lipid peroxides. The loss of this pathway can lead to lethality of mouse embryos at E7.5 stage, and tissue-specific knockout can also cause embryonic developmental defects. SSMD (somatic cell mitochondrial dysfunction) links maternal GPX4 expression to blastocyst quality. **(B)** Ferroptosis suppressor protein1 (FSP1)/coenzyme Q10 (CoQ10)-mediated non-glutathione-dependent compensation pathway: FSP1 catalyzes the reduction of CoQ10 to ubiquinol (CoQ10H_2_) to independently inhibit ferroptosis. Abnormal function of FSP1 can impair placental angiogenesis and embryo implantation ability. It is an indispensable backup regulatory pathway during embryonic development. **(C)** Iron metabolism and lipid peroxidation regulatory network: Imbalance in iron absorption, storage and polyunsaturated fatty acids (PUFAs) metabolism can directly drive lipid peroxidation and ferroptosis. NARFL (nuclear assembly factor 1-like, also known as CIAPIN1), ACO1 (aconitase 1, also known as IRP1 or iron regulatory protein 1), and IRP1 are key components of the cytoplasmic iron-sulfur cluster assembly (CIA) system and iron regulatory network. HMOX1 (heme oxygenase 1) and TXNIP (thioredoxin-interacting protein) are involved in iron metabolism regulation and oxidative stress response. Disorders of this network have been shown to be associated with embryonic developmental arrest and pregnancy complications in early pig embryos and zebrafish models.

Ferroptosis is essentially iron-catalyzed lipid peroxidation of cell membranes, generating toxic lipid peroxides that damage membrane integrity and ultimately lead to cell death (Zheng et al., 2024). The classical antioxidant pathway GPX4-GSH is a core defense barrier in the body that blocks the cascade of ferroptosis, and its functional integrity directly determines the fate of embryonic development (You et al., 2024) (Figure 2A). This pathway, centered on GPX4, utilizes GSH as a specific reducing agent to reduce toxic lipid peroxides to non-toxic alcohols, thus breaking the chain reaction of lipid peroxidation at its source, maintaining cell membrane integrity, and blocking ferroptosis (Dar et al., 2024). Among cellular antioxidant enzymes, only GPX4 possesses the function of directly reducing lipid peroxides, making it irreplaceable (Shen et al., 2025). It is the core molecule of the ferroptosis inhibition pathway, and its activity directly determines the cell’s sensitivity to ferroptosis, precisely regulating the dynamic balance between cell survival and death (Dai et al., 2024).

The regulation of ferroptosis by the GPX4-GSH pathway is a dynamic process of “defense-imbalance-amplification”: Under physiological conditions, GPX4 continuously scavenges lipid peroxides using GSH as a substrate, controlling oxidative stress within the physiological threshold and maintaining redox homeostasis required for embryonic development (Nakamura and Takada, 2021). When pathological factors (such as iron overload, GSH depletion, and GPX4 expression downregulation) impair pathway function, GPX4’s antioxidant capacity is weakened, and lipid peroxides that cannot be cleared in time accumulate intracellularly. This further exacerbates the iron redox cycle through the Fenton reaction, forming a positive feedback cascade amplification loop of “GPX4 function inhibition → lipid peroxide accumulation → increased iron overload → further decrease in GPX4 activity,” ultimately triggering large-scale ferroptosis and leading to embryonic developmental arrest (Du et al., 2022). However, it should be noted that ferroptosis can also be directly induced by GPX4 inhibition, cysteine deprivation, or ACSL4 upregulation, without necessarily requiring prior iron overload as an initiating factor. GPX4 is crucial for embryonic development, directly regulating embryonic survival and normal development (Xie et al., 2023). The integrity and sensitivity of this pathway are fundamental to the embryo’s adaptation to the redox needs of different developmental stages: cells precisely maintain a dynamic balance between survival and death through differentiated antioxidant pathway configuration and sensitivity, ensuring normal early embryonic development, further confirming that this pathway is an indispensable core regulatory pathway for early embryonic development (Wilkinson et al., 2023).

Studies show that naïve pluripotent mouse embryonic stem cells have low GPX4 expression levels and lack compensatory antioxidant pathways, making them highly sensitive to GPX4 inhibition-induced ferroptosis: when GPX4 activity is inhibited, GSH cannot be effectively utilized, and lipid peroxides cannot be cleared, instantly triggering the ferroptosis cascade amplification loop, leading to rapid death of embryonic stem cells; while systemic knockout mice of the GPX4 gene die at 7.5 days of embryonic development (E7.5), directly confirming that GPX4 is an absolutely essential molecule for blocking cascade amplification and maintaining embryonic survival, and its absence directly leads to uncontrolled ferroptosis cascade amplification, causing embryonic lethality (Lee et al., 2023). Nevertheless, whether the embryonic lethality in GPX4 systemic knockout mice is exclusively attributable to ferroptosis remains debated, as GPX4 deficiency may concurrently activate other cell death pathways such as apoptosis or necrosis. The aforementioned differences essentially reflect the varying defense thresholds of embryonic cells against the amplified ferroptosis cascade: Naïve pluripotent mouse embryonic stem cells, due to insufficient GPX4 expression and the lack of compensatory pathways, have an extremely low “initiation threshold” for the cascade amplification, allowing even mild oxidative stress to trigger a chain reaction (Chen et al., 2023). In contrast, human embryonic stem cells, through haigh GPX4 expression and the construction of a multi-pathway defense network, significantly increase the initiation threshold of the cascade amplification, thereby acquiring stronger ferroptosis resistance (Gao and Du, 2025). This threshold regulation is the core mechanism by which embryos adapt to the redox needs of different developmental stages and forms the structural basis for the dual physiological effects of ferroptosis (Chen et al., 2021a).

Studies show that naïve pluripotent mouse embryonic stem cells, with low GPX4 expression and a lack of compensatory antioxidant pathways, are highly sensitive to GPX4-inhibited ferroptosis; while initiated human embryonic stem cells, with sufficient GPX4 expression and multiple backup pathways, exhibit relatively stronger ferroptosis resistance (Ma et al., 2022). The comparative results of the two stem cell types validate these findings. Furthermore, the significant upregulation of GPX4 transcripts and genes related to oxidative phosphorylation in the gestational sac cell mass suggests a higher metabolic rate in early embryos, consistent with the principle that “the more vigorous the metabolism, the more oxidants are produced (Deluao et al., 2022).” This confirms the conclusion that early embryos are highly dependent on the antioxidant defense system to maintain redox balance, echoing the view that redox needs vary at different cellular stages (Divvela et al., 2025). It is worth noting that these comparative conclusions are primarily derived from *in vitro* cell culture models and animal experiments, and direct extrapolation from mouse embryonic stem cells to human embryonic development requires careful validation due to potential interspecies differences in redox regulation and ferroptosis sensitivity.

Studies have shown that maternal smoking during pregnancy can lead to hypermethylation of the GPX4 gene in neonatal umbilical cord blood, inhibiting GPX4 expression, weakening its antioxidant pathway function, and preventing effective inhibition of ferroptosis, ultimately leading to abnormal cell death and fetal growth restriction. Therefore, external environmental factors can damage normal embryonic development by interfering with GPX4-dependent antioxidant pathways.

In summary, GPX4-dependent antioxidant pathways are not only vital for early embryonic development, but their homeostasis imbalance is also a key mechanism inducing developmental abnormalities, providing a core target for embryonic development regulation and birth defect prevention (Ufer et al., 2010).

### 2.2 FSP1-CoQ10 glutathione-independent antioxidant compensation pathway

This pathway can terminate the lipid peroxidation chain reaction, inhibiting ferroptosis at its source (Fujii and Yamada, 2023). Simultaneously, ubiquinol (CoQ10H_2_) protects the integrity of the mitochondrial membrane structure, preventing oxidative damage to mitochondrial DNA and maintaining normal mitochondrial energy supply (Huang et al., 2020). The normal operation of the classic glutathione-dependent antioxidant pathway of GPX4-GSH requires energy support, and stable mitochondrial energy supply further ensures the redox balance of the GPX4-GSH pathway (Su et al., 2026). The cascade amplification of ferroptosis is essentially a positive feedback loop driven by lipid peroxidation, while the ferroptosis suppressor protein1 (FSP1)-coenzyme Q10 (CoQ10) pathway is a key compensatory defense barrier independent of GPX4-GSH: FSP1 uses NAD(P)H as a coenzyme to reduce CoQ10 to ubiquinol (CoQ10H_2_) (Li P. et al., 2025) (Figure 2B). Ubiquinol (CoQ10H_2_) can directly scavenge lipid peroxide free radicals on the cell membrane, cutting off the initiation of the cascade amplification at its source (Li H. et al., 2023). Simultaneously, ubiquinol (CoQ10H_2_) stabilizes mitochondrial membrane localization, protects mitochondrial structural integrity, and blocks the release of iron ions and reactive oxygen species (ROS) bursts caused by mitochondrial damage, inhibiting the cascade amplification from a secondary driving point, thus constructing a dual-line ferroptosis defense system of “source blocking + mitochondrial protection (She et al., 2023).” While the FSP1-CoQ10 glutathione-independent antioxidant compensation pathway complements the GPX4-GSH pathway, its physiological function is equally indispensable, assisting the classical GPX4-GSH antioxidant pathway in its function during embryonic development (Wang et al., 2026). In summary, the FSP1-CoQ10 glutathione-independent antioxidant compensation pathway can independently inhibit ferroptosis while simultaneously assisting the classical GPX4-GSH pathway in maintaining ferroptosis homeostasis (Miao et al., 2022).

Studies have confirmed that CoQ10, as a core substrate of this pathway, requires a specific rate-limiting enzyme for its synthesis *in vivo*; a deficiency in this enzyme directly leads to insufficient CoQ10 synthesis, preventing pathway activation, depriving cells of their antioxidant reserve, and inducing ferroptosis (Kagan et al., 2024). Vascular cell membranes are rich in lipids, and lipid peroxidation is the core of ferroptosis (Endale et al., 2023). This process directly damages the integrity of vascular cell membranes, causing structural damage and functional abnormalities in blood vessels, ultimately leading to impaired embryonic vascular development, reduced vascular density, and lethality in embryos (Hermann et al., 2021). In zebrafish models, hepcidin gene knockout has been employed to investigate iron metabolism in embryonic development, though the resulting phenotypic outcomes—often characterized by iron overload rather than pronounced developmental delay—may differ substantially from those observed in mammalian systems.

In early porcine embryonic development models, FSP1 is expressed at all developmental stages, suggesting the important role of this pathway in embryonic development (Wang Y. Q. et al., 2024). Given its close connection with the vascular system, the entire embryonic development process relies on the FSP1-CoQ10 glutathione-independent antioxidant compensation pathway to maintain vascular structural and functional stability (Li W. et al., 2023). Inhibition of FSP1 expression directly leads to the blockage of this pathway, failing to effectively block lipid peroxidation, inducing ferroptosis, and causing vascular structural damage (Chen et al., 2021b). Simultaneously, cell membrane damage interferes with cell division, reducing embryonic cleavage rate and blastocyst formation rate, ultimately resulting in decreased blastocyst quality (Yazdani et al., 2024). These results confirm that even though the FSP1-CoQ10 glutathione-independent antioxidant compensation pathway is a substitute pathway, it plays an irreplaceable and crucial role in early embryonic development (Wang D. et al., 2024). Furthermore, the classical GPX4-GSH antioxidant pathway and the FSP1-CoQ10 glutathione-independent antioxidant compensation pathway complement and synergistically work together (Artusi et al., 2025). Relying solely on the FSP1-CoQ10 glutathione-independent antioxidant compensation pathway cannot fully compensate for the functional deficiencies of the classical GPX4-GSH pathway; their synergy is the core guarantee for normal embryonic development. The GPX4-GSH and FSP1-CoQ10 pathways construct a dual-blocking network against ferroptosis cascade amplification through a synergistic mechanism of “core blockade of the main pathway + redundant defense of the compensatory pathway”: the GPX4-GSH pathway acts as the main defense, responsible for continuously clearing basal lipid peroxides and blocking cascade amplification at the core stage; the FSP1-CoQ10 pathway acts as a compensatory defense, being activated compensatorily when GPX4 function is impaired, preventing uncontrolled cascade amplification. This dual-pathway synergistic mechanism is the core guarantee for maintaining ferroptosis homeostasis and oxidative stress fluctuations during embryonic adaptation and development, and also an important structural basis for the dual physiological effects of ferroptosis.

### 2.3 Physiological ferroptosis in embryonic development: beyond pathological cell death

While the pathological role of ferroptosis in embryonic development has been extensively discussed above, the physiological functions of regulated ferroptosis in normal developmental processes deserve equal attention. The concept of “trigger waves”—spatiotemporally coordinated cell death events that propagate across tissues—has emerged as a critical mechanism for tissue sculpting during embryonic development. For example, in avian embryogenesis, iron-dependent trigger waves mediate limb muscle remodeling through spatially restricted ferroptotic cell clearance, where precisely regulated ferroptosis removes excess cells to shape tissue architecture. It should be clarified that interdigital cell clearance is classically mediated by apoptosis, not ferroptosis. Similarly, in avian limb development, localized ferroptosis contributes to tissue remodeling and digit separation, demonstrating that programmed cell death via the ferroptosis pathway is not merely a pathological outcome but an integral component of morphogenetic programs.

These physiological ferroptosis events are tightly integrated with canonical morphogenetic signaling pathways, including Bone Morphogenetic Protein (BMP), Fibroblast Growth Factor (FGF), and Hedgehog signaling. For instance, BMP signaling can modulate cellular iron sensitivity and lipid metabolism, thereby influencing the threshold for ferroptosis initiation in specific tissue domains. FGF signaling regulates the expression of antioxidant enzymes including GPX4 and SLC7A11, creating regional differences in ferroptosis resistance that guide tissue patterning. The cross-talk between Hedgehog signaling and iron metabolism—particularly through the regulation of iron transport and storage genes—further underscores the embeddedness of ferroptosis regulation within developmental signaling networks. Thus, ferroptosis should be viewed not only as a pathological cascade to be suppressed, but as a developmentally regulated process whose precise spatiotemporal control is essential for normal embryonic morphogenesis.

### 2.4 Cascade-like damage from iron metabolism homeostasis imbalance and precise clinical intervention

Imbalance in iron metabolism is a core driving factor in multi-system pathological damage, and its harm has a cascading amplification effect. Abnormal accumulation of iron ions significantly amplifies the lipid peroxidation chain reaction through the Fenton reaction, exacerbating oxidative stress, damaging cell membrane integrity, and causing ion disorder, directly damaging vascular development and threatening embryonic survival (Ammendolia et al., 2021). Iron homeostasis is the core initiator of the ferroptosis cascade: Under physiological conditions, cells maintain free ferrous ions at extremely low levels through strict regulation of iron transport, storage, and utilization, blocking the initiation of the Fenton reaction and lipid peroxidation at the source (He et al., 2020). When iron homeostasis is imbalanced, free ferrous ions accumulate in large quantities, catalyzing the generation of (·OH) through the Fenton reaction, initiating initial lipid peroxidation. The generated LPO not only directly damages the cell membrane but also continuously consumes GSH and inhibits GPX4 activity, while simultaneously disrupting the mitochondrial protective function of the FSP1-CoQ10 pathway, completely dismantling the body’s dual antioxidant defense system (Zhu et al., 2024) (Figure 2C). After the defense system collapses, LPO cannot be cleared, further aggravating the redox cycle of iron ions, rapidly amplifying initial micro-damage into multi-system devastating damage (Liew and Kubes, 2019). This pathological process can simultaneously destroy the synergistic defense system of the GPX4-GSH-dependent classical antioxidant pathway and the FSP1-CoQ10-independent antioxidant compensatory pathway, completely dismantling the cell’s oxidative stress buffering capacity and amplifying the initial micro-damage cascade into devastating tissue damage (Yu et al., 2022).

Ferroptosis does not operate in isolation during embryonic development; rather, it exhibits extensive cross-talk with other programmed cell death modalities, including apoptosis, autophagy (particularly ferritinophagy, a selective autophagy process mediated by NCOA4 that degrades ferritin to release free iron and promote ferroptosis), cuproptosis (copper-dependent regulated cell death), and necrosis. These cell death pathways are not mutually exclusive but form an interconnected network: ferroptosis and apoptosis can share common regulatory nodes such as p53 and BCL-2 family proteins, and the inhibition of one pathway may redirect cells toward alternative death mechanisms. Ferritinophagy directly links autophagy to ferroptosis by controlling intracellular iron availability, representing a critical convergence point between autophagic and ferroptotic pathways. The interaction between ferroptosis and cuproptosis is worth noting. ATP7A deficiency causes Menkes disease. Menkes disease is a copper transport disorder. However, ATP7A deficiency mainly leads to iron dyshomeostasis. This increases ferroptosis susceptibility. The copper-iron metabolic link is complex. In embryonic cell death, iron dyshomeostasis is the key factor. Direct copper-mediated cell death is secondary. The precise mechanism still needs clarification. Animal experiments have confirmed that the loss of function of key iron transport proteins blocks iron cycling, exacerbates the pathological accumulation of iron ions, and further amplifies the damage effect (Ru et al., 2024). In this context, NARFL (nuclear assembly factor 1-like, also known as CIAPIN1 or cytosolic iron-sulfur cluster assembly factor), ACO1 (aconitase 1, also known as IRP1 or iron regulatory protein 1), and IRP1 itself represent critical components of the iron-sulfur cluster assembly machinery and iron regulatory network. Deficiency in the cytoplasmic iron-sulfur cluster assembly system, such as NARFL defect, leads to loss of ACO1 enzyme function, which in turn disrupts IRP1-mediated iron regulation, causing intracellular iron overload, endothelial cell ferroptosis, and ultimately embryonic vascular defects and lethality. Fetal iron metabolism depends entirely on maternal iron transport supply via the umbilical cord; maternal iron homeostasis imbalance can directly interfere with fetal iron metabolism regulation, significantly increasing the risk of embryonic lethality (Zhang et al., 2022). The damage caused by iron homeostasis imbalance has a diffusion characteristic from the microscopic to the macroscopic, and can comprehensively disrupt the normal physiological functions of multiple tissues and systems.

Clinical intervention needs to precisely target the root cause of the imbalance, abandon ineffective remedies for secondary links, quickly stabilize iron balance, block the cascading damage of oxidative stress, effectively improve the patient’s physical condition, and provide a clear and feasible plan for clinical treatment (Plascencia-Villa and Perry, 2021). The core of clinical intervention lies in precisely disrupting the key nodes of the cascade circuit: regulating iron ion accumulation by targeting iron transport proteins, supplementing with antioxidants to block lipid peroxidation, and activating the GPX4/FSP1 pathway to rebuild the defense system. This approach blocks the cascade amplification from multiple stages—from the source to the middle and end—rather than simply treating downstream damage. However, the clinical application of ferroptosis inhibitors during pregnancy requires careful consideration of their specificity and potential off-target effects. Many currently available ferroptosis inhibitors may exhibit non-specific antioxidant activities or interfere with other redox-sensitive developmental processes, raising concerns about unintended consequences for normal embryonic development. This precise intervention strategy provides a new theoretical basis and practical direction for the prevention and treatment of embryonic developmental abnormalities and birth defects related to iron metabolism imbalance (Kakebeen and Niswander, 2021).

## 3 Multisystemic pathological effects and clinical significance of ferroptosis amplification effect

### 3.1 The regulatory role of ferroptosis cascade amplification in nervous system development and remodeling

**FIGURE 3:**
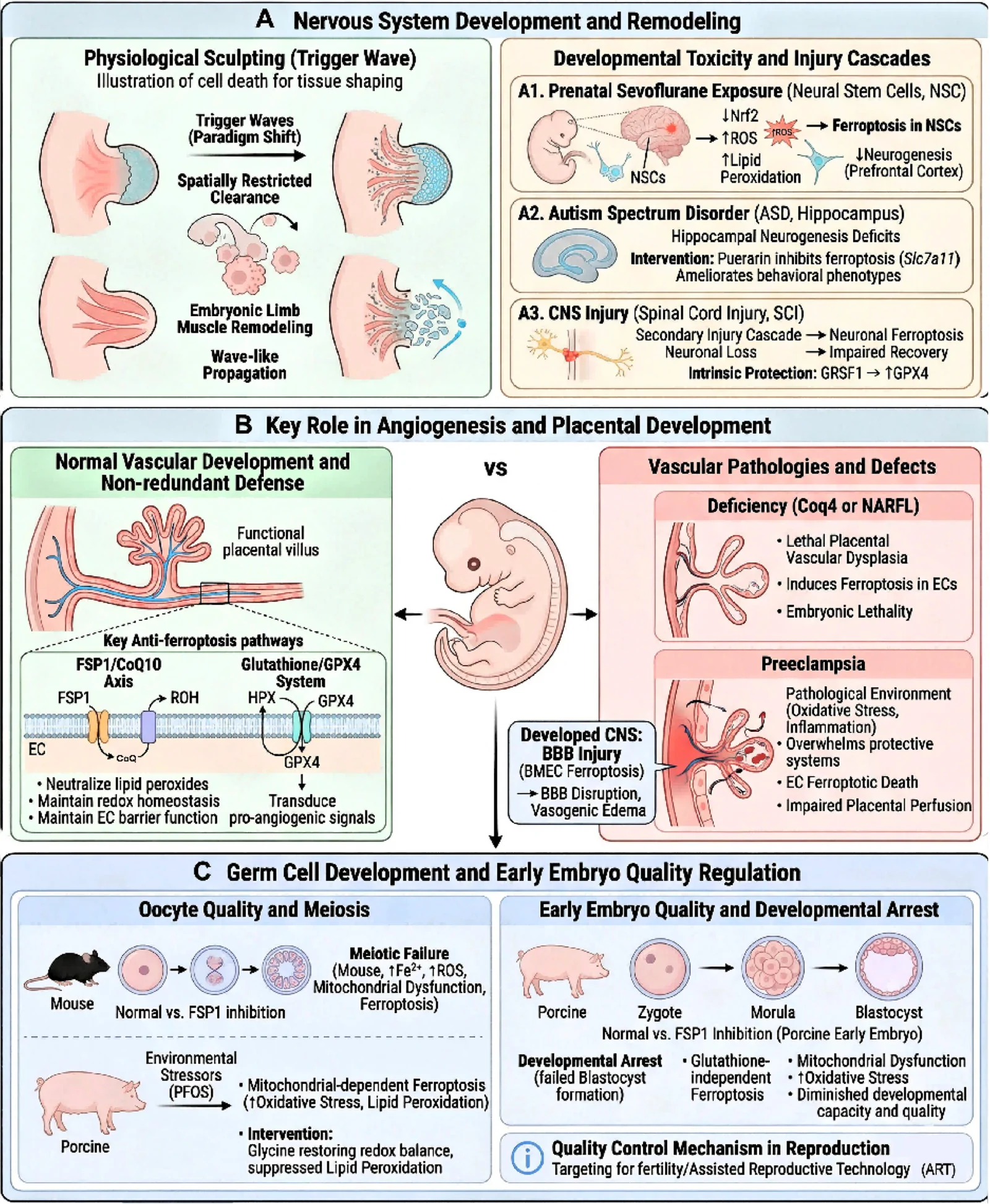
The role of ferroptosis in key stages of early embryonic development. **(A)** Nervous system development and remodeling:The left panel illustrates a physiological example of ferroptotic trigger waves in avian embryonic limb muscle remodeling (a tissue-sculpting process mediated by ferroptosis, distinct from interdigital cell clearance which is apoptosis-dependent). The right panel depicts pathological conditions in the nervous system. Under physiological conditions, iron-dependent trigger waves coordinate spatially restricted cell clearance and embryonic limb muscle remodeling through wave-like propagation of programmed cell death, participating in tissue sculpting and nervous system shaping. Under pathological conditions, abnormal ferroptosis can lead to nerve cell damage. Prenatal sevoflurane exposure induces ferroptosis in neural stem cells (NSCs) through NRF2 downregulation, ROS elevation, and lipid peroxidation, resulting in impaired neurogenesis in the prefrontal cortex. In autism spectrum disorder (ASD), hippocampal neurogenesis deficits occur; puerarin inhibits ferroptosis by targeting SLC7A11 and ameliorates behavioral phenotypes. In central nervous system injury (spinal cord injury, SCI), secondary injury cascades trigger neuronal ferroptosis and neuronal loss, impairing recovery; intrinsic protection through GRSF1-mediated GPX4 upregulation provides a protective mechanism. **(B)** Angiogenesis and embryonic development: The GPX4-dependent glutathione pathway and the ferroptosis suppressor protein 1 (FSP1)-coenzyme Q10 (CoQ10) pathway constitute key anti-ferroptosis axes in endothelial cells (ECs), neutralizing lipid peroxides, maintaining redox homeostasis, maintaining EC barrier function, and transducing pro-angiogenic signals. Deficiency in Coq4 (coenzyme Q4 biosynthesis protein) or NARFL (nuclear assembly factor 1-like, also known as CIAPIN1) leads to lethal placental vascular dysplasia, induces ferroptosis in ECs, and causes embryonic lethality. In preeclampsia, pathological environments characterized by oxidative stress and inflammation overwhelm protective systems, resulting in EC ferroptotic death and impaired placental perfusion. In the developed central nervous system, blood-brain barrier (BBB) injury (BMEC ferroptosis) leads to BBB disruption and vasogenic edema. **(C)** Germ cell and early embryo quality regulation: Ferroptosis homeostasis is closely related to oocyte quality and early embryonic development. In mouse oocytes, FSP1 inhibition leads to meiotic failure characterized by elevated Fe^2+^, increased ROS, mitochondrial dysfunction, and ferroptosis. In porcine models, environmental stressors such as PFOS induce mitochondrial-dependent ferroptosis through oxidative stress and lipid peroxidation; glycine intervention restores redox balance and suppresses lipid peroxidation. In porcine early embryos, normal development versus FSP1 inhibition results in developmental arrest (failed blastocyst formation) through glutathione-independent ferroptosis, accompanied by mitochondrial dysfunction, elevated oxidative stress, and diminished developmental capacity and quality. These quality control mechanisms in reproduction represent potential targets for fertility improvement and assisted reproductive technology (ART).

Iron metabolism homeostasis directly regulates neural stem cell development, and its balance determines the intensity of ferroptosis damage (Yang et al., 2024). Excessive accumulation of iron ions continuously induces high-intensity oxidative stress, which in turn directly leads to abnormal function of the nuclear factor erythroid 2-related factor 2 (NRF2) transcription factor, preventing it from properly regulating the synthesis of antioxidant proteins, thus amplifying the ferroptosis damage effect in a cascade (Punziano et al., 2024). The nervous system, due to its cell membranes being rich in polyunsaturated fatty acids and its vigorous oxidative metabolism, is highly sensitive to the amplification of ferroptosis cascades. Its pathological process exhibits a “start-amplification-diffusion” characteristic: excessive iron accumulation initiates initial lipid peroxidation through the Fenton reaction; the generated LPO directly attack the nerve cell membrane, while simultaneously inhibiting the nuclear translocation and transcriptional activity of NRF2 (Zhang et al., 2020) (Figure 3A). After NRF2 inactivation, the expression of core antioxidant enzymes such as GPX4 and FSP1 is significantly downregulated, completely dismantling the nerve cell’s dual antioxidant defense system. With the defense system collapsing, LPO cannot be cleared, further exacerbating the iron redox cycle, rapidly amplifying initial oxidative damage into large-scale nerve cell ferroptosis (Qiang et al., 2026).

It is important to clarify that while certain clinical anesthetics (such as sevoflurane) have been shown to disrupt iron metabolic homeostasis and exacerbate oxidative stress in experimental settings, the clinical use of anesthetics does not rely on ferroptosis induction as a mechanism for achieving neural inhibition. The primary mechanism of clinical anesthesia involves the modulation of neurotransmitter receptors and ion channels, not the targeted induction of ferroptosis. The observed ferroptosis-related damage associated with anesthetic exposure in experimental models represents an off-target toxic effect rather than a therapeutic mechanism. Therefore, the notion that ferroptosis induction can be harnessed for “controllable neural inhibition” to improve anesthetic precision is scientifically inaccurate and should be reframed as a risk factor for neurotoxicity rather than a therapeutic strategy.

Ferroptosis is not a single-cell injury; the damage signal propagates in a wave-like manner, triggering widespread nerve cell death with extremely strong destructive effects. Impaired nerve function directly blocks motor signal transmission, severely affecting the body’s normal motor abilities. Precise regulation of iron metabolic homeostasis can inhibit abnormal ferroptosis, clear diseased nerve cells, and promote the recovery of nerve and muscle function. Maintaining iron metabolic balance can optimize neural development and functional construction, providing clear and practical theoretical support for understanding anesthesia-related neurotoxicity and developing protective strategies and treating nervous system diseases (Kulaszyńska et al., 2024).

### 3.2 CoQ10 synthesis defects mediate inactivation of dual antioxidant pathways: ferroptosis cascade amplification circuit and endothelial-multi-system damage mechanism

Imbalance in iron metabolism is a core trigger for endothelial cell ferroptosis, which can directly disrupt the integrity of vascular endothelial cell membranes and severely impair endothelial cell survival, migration, and angiogenesis (Shi et al., 2024). Defects in key genes involved in CoQ10 synthesis significantly amplify endothelial ferroptosis damage: CoQ10, as a core cofactor in the FSP1-CoQ10-independent antioxidant compensation pathway, directly weakens the cell membrane’s antioxidant defense capabilities, further exacerbating endothelial damage (Zha et al., 2023). The core driving force behind the ferroptosis cascade amplification is a chain reaction of “collapse of the alternative defense → uncontrolled oxidative stress → inactivation of the classical pathway” (Figure 3B), forming a closed-loop cascade amplification loop, rapidly amplifying the initial endothelial micro-damage into structural and functional destruction of the entire vascular system (Wawrzyniak and Balawender, 2022). Such gene abnormalities can form a positive feedback loop of “iron metabolism disorder - oxidative stress amplification - endothelial damage”, which promotes the rapid spread of damage and causes a sudden failure of multiple system functions (Jipeng et al., 2026).

In this context, NARFL (nuclear assembly factor 1-like, also known as CIAPIN1 or cytosolic iron-sulfur cluster assembly factor) and Coq4 (coenzyme Q4 biosynthesis protein) represent critical genes whose deficiency disrupts both the cytoplasmic iron-sulfur cluster assembly system and CoQ10 synthesis, respectively. Deficiency in NARFL leads to loss of ACO1 (aconitase 1, also known as IRP1 or iron regulatory protein 1) enzyme function, disrupting IRP1-mediated iron regulation and causing intracellular iron overload, endothelial cell ferroptosis, and embryonic vascular defects and lethality (Figure 3B).

### 3.3 Pathological mechanisms and clinical intervention strategies of reproductive system damage mediated by ferroptosis cascade amplification loop

The cascade effect of ferroptosis is a core driving mechanism for reproductive and developmental abnormalities (Ye et al., 2025). Imbalances in iron metabolism homeostasis directly damage germ cells, the endometrium, and placental vascular endothelium through continuous oxidative stress, disrupting the maternal-fetal material exchange barrier and significantly increasing the risk of embryonic lethality, recurrent miscarriage, and intrauterine growth retardation (Lu et al., 2025). The reproductive system is highly sensitive to oxidative stress. Ferroptosis cascade amplification in this system presents a pathological process of “initiation at the source - multi-stage amplification - terminal damage” (Figure 3C), forming a closed-loop positive feedback loop, rapidly amplifying local oxidative damage into systemic damage at the maternal-fetal interface, ultimately leading to abnormal embryonic development and pregnancy failure (Zhang C. et al., 2023).

Clinical intervention should follow the principle of “source blocking - pathway regulation - precise monitoring”: iron overload should be corrected by iron chelators, the classical antioxidant pathway dependent on GPX4-GSH and the antioxidant compensation pathway independent of FSP1-CoQ10 should be targeted and activated, the NRF2 antioxidant system should be stabilized, and the damage cascade amplification should be blocked; at the same time, individualized iron metabolism and oxidative stress monitoring should be combined to achieve precise prevention and treatment of reproductive and developmental diseases, and provide clear and feasible clinical solutions for improving pregnancy outcomes (Auerbach, 2023).

## 4 The regulation of ferroptosis cascade amplification by environmental and drug factors and its maternal pathological effects

### 4.1 Induction and intervention targets of environmental pollutants in the amplification of ferroptosis cascade

**FIGURE 4:**
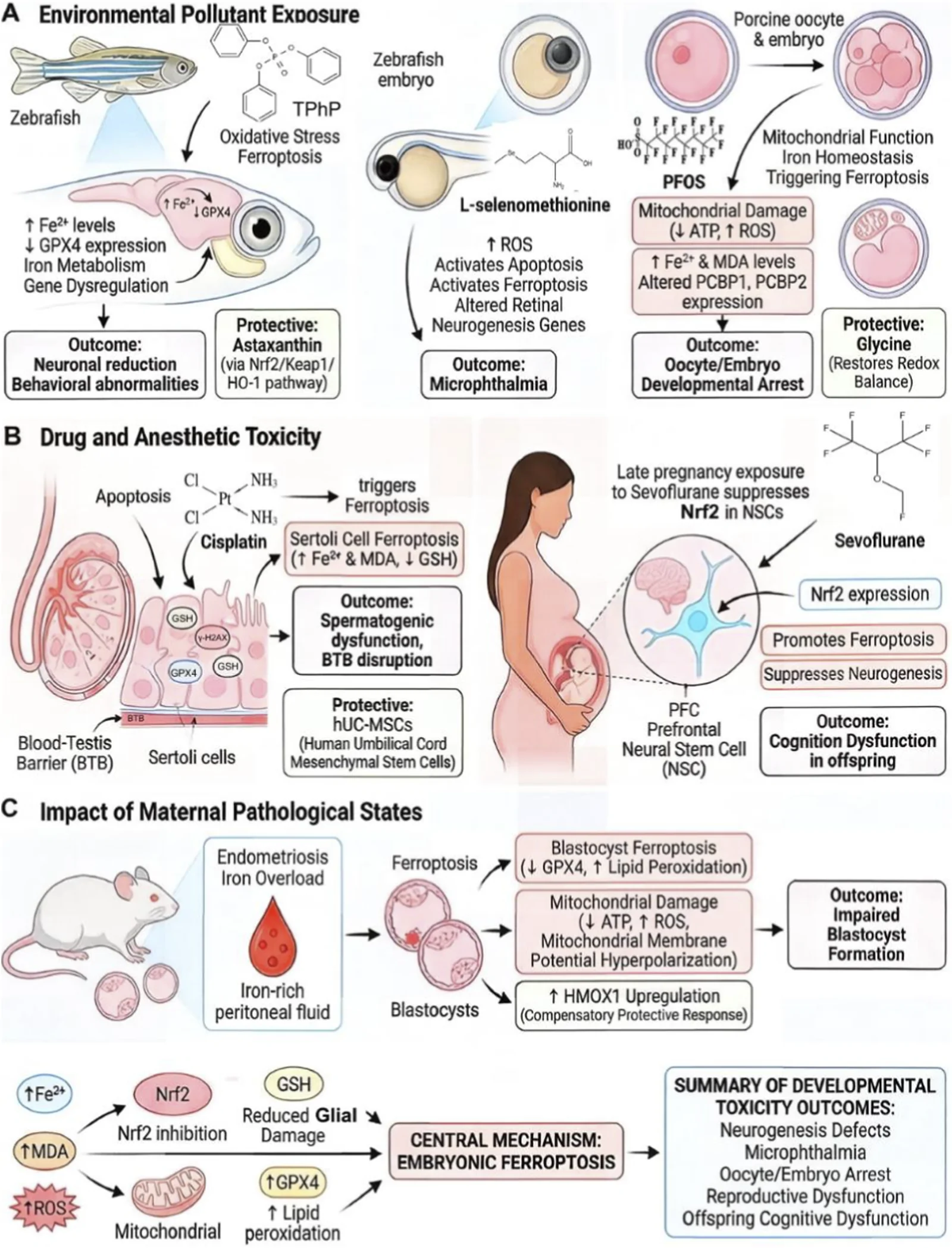
Key mechanisms by which environmental factors and maternal pathological states regulate embryonic ferroptosis. **(A)** Exposure to environmental pollutants: Environmental pollutants can directly trigger embryonic ferroptosis by inducing oxidative stress and interfering with iron metabolism and the function of the glutathione peroxidase 4 (GPX4)-dependent antioxidant pathway. In zebrafish models, triphenyl phosphate (TPhP) exposure elevates Fe^2+^ levels, downregulates GPX4 expression, and causes iron metabolism gene dysregulation, resulting in neuronal reduction and behavioral abnormalities; astaxanthin provides protection via the NRF2/Keap1/HO-1 pathway. L-selenomethionine exposure increases ROS, activates apoptosis and ferroptosis, alters retinal neurogenesis genes, and leads to microphthalmia. In porcine oocyte and embryo models, perfluorooctane sulfonate (PFOS) exposure causes mitochondrial damage characterized by decreased ATP and increased ROS, elevated Fe^2+^ and MDA levels, altered PCBP1 and PCBP2 expression, and ultimately oocyte/embryo developmental arrest; glycine intervention restores redox balance and provides protective effects. **(B)** Drug and anesthetic toxicity: Exogenous stimuli such as cisplatin (CDDP) and pregnancy anesthetics can directly activate ferroptosis signals, damage germ cells and embryonic development potential, and cause sperm dysfunction, embryonic development defects and offspring neurocognitive dysfunction. Cisplatin triggers Sertoli cell ferroptosis characterized by elevated Fe^2+^ and MDA levels and depleted GSH, leading to spermatogenic dysfunction and blood-testis barrier (BTB) disruption; human umbilical cord mesenchymal stem cells (hUC-MSCs) provide protective effects. Late pregnancy exposure to sevoflurane suppresses NRF2 expression in neural stem cells (NSCs), promotes ferroptosis, suppresses neurogenesis in the prefrontal cortex (PFC), and results in offspring cognition dysfunction. **(C)** Impact of maternal pathological conditions: Maternal pathological conditions such as endometriosis and iron overload can induce embryonic ferroptosis by increasing oxidative stress levels and inhibiting the glutathione (GSH) system, leading to blastocyst mitochondrial damage and implantation failure, thus affecting pregnancy outcomes. In endometriosis-associated infertility, iron-rich peritoneal fluid triggers blastocyst ferroptosis characterized by GPX4 downregulation, elevated lipid peroxidation, mitochondrial damage (decreased ATP, increased ROS, mitochondrial membrane hyperpolarization), and HMOX1 (heme oxygenase 1) upregulation as a compensatory protective response, ultimately resulting in impaired blastocyst formation. The central mechanism converges on embryonic ferroptosis, with developmental toxicity outcomes including neurogenesis defects, microphthalmia, oocyte/embryo arrest, reproductive dysfunction, and offspring cognitive dysfunction.

Multiple environmental pollutants mediate multisystemic developmental toxicity by disrupting iron metabolism homeostasis and triggering a cascade effect of ferroptosis (Cao et al., 2025). Aspartame induces iron overload and lipid peroxidation, triggering ferroptosis, inhibiting embryonic proliferation, and causing abnormal pigmentation. Dibutyl phthalate (DBP) induces neuronal ferroptosis, inhibits the expression of neurodevelopmental genes and the release of neurotransmitters, causing motor dysfunction. The iron chelator deferasirox can effectively reverse this damage. Perfluorooctane sulfonate (PFOS) damages germ cells through the ferroptosis pathway, interfering with gamete maturation and embryonic development, and producing reproductive toxicity (Sun et al., 2025) (Figure 4A). In zebrafish models, triphenyl phosphate (TPhP) exposure induces oxidative stress and ferroptosis, leading to elevated Fe^2+^ levels, downregulated GPX4 expression, iron metabolism gene dysregulation, neuronal reduction, and behavioral abnormalities; L-selenomethionine treatment can activate apoptosis and ferroptosis, alter retinal neurogenesis genes, and result in microphthalmia. In porcine oocyte and embryo models, PFOS exposure causes mitochondrial damage characterized by decreased ATP and increased ROS, elevated Fe^2+^ and MDA levels, altered PCBP1 and PCBP2 expression, and ultimately oocyte/embryo developmental arrest; glycine intervention restores redox balance and provides protective effects (Figure 4A).

Environmental pollutants, acting as exogenous stressors, amplify toxicity through a multi-stage targeted disruption of the homeostatic regulation of the ferroptosis cascade amplification loop. The core pathogenic mechanism involves initiating and accelerating a positive feedback loop of lipid peroxidation at its source. This is achieved by inducing abnormal iron accumulation, directly inhibiting the activity of the classical GPX4-GSH antioxidant pathway and the FSP1-CoQ10-independent antioxidant compensation pathway, and downregulating the expression and function of NRF2 and its downstream antioxidant pathways, thus completely dismantling the cell’s oxidative defense barrier. After the defense system collapses, lipid peroxides cannot be promptly cleared, further promoting the iron redox cycle, forming a complete cascade amplification loop. This rapidly amplifies local damage into multisystemic developmental toxicity. Different pollutants achieve targeted toxicity by acting on different nodes of this loop, while iron chelators and antioxidants can reverse damage by blocking key links in the cascade amplification, providing clear molecular targets for the prevention and treatment of environment-related developmental abnormalities (Gao L. et al., 2026).

### 4.2 The regulatory effect of anesthetic and related drugs on the amplification of the ferroptosis cascade

Drugs and anesthetics can induce ferroptosis by disrupting iron homeostasis, mediating multi-system toxicity (Li W. et al., 2025). Cisplatin (CDDP) induces iron-dependent lipid peroxidation and oxidative stress, damaging the reproductive barrier and causing germ cell dysfunction and infertility; sevoflurane disrupts the iron and mitochondrial balance in neural stem cells, triggering ferroptosis, inhibiting neurogenesis, and causing neurodevelopmental and cognitive impairment. Ferroptosis provides a core mechanistic basis for understanding medical-related risks and protective strategies (Zhou et al., 2024) (Figure 4B). In detail, cisplatin triggers Sertoli cell ferroptosis characterized by elevated Fe^2+^ and MDA levels and depleted GSH, leading to spermatogenic dysfunction and blood-testis barrier (BTB) disruption; human umbilical cord mesenchymal stem cells (hUC-MSCs) provide protective effects. Late pregnancy exposure to sevoflurane suppresses NRF2 expression in neural stem cells (NSCs), promotes ferroptosis, suppresses neurogenesis in the prefrontal cortex (PFC), and results in offspring cognition dysfunction (Figure 4B).

As exogenous stressors, anesthetic and chemotherapy drugs exhibit a core toxicity mechanism that strictly follows the classic pathway of ferroptosis cascade amplification. This rapidly amplifies the initial drug toxicity into irreversible damage to multiple systems, providing core mechanistic support for understanding medical-related risks and targeted protection strategies (Kang et al., 2021).

### 4.3 The influence of maternal pathological conditions on fetal ferroptosis and development

Maternal pathological conditions alter the uterine microenvironment, triggering iron metabolism disorders and oxidative stress, inducing embryonic ferroptosis, and interfering with embryonic nutrient supply and development (Wang et al., 2025). Ferroptosis amplifies, forming a positive feedback loop of “iron disorder-ferroptosis-inflammation,” continuously exacerbating intrauterine environment damage and significantly increasing the risk of adverse pregnancy outcomes (Thornton et al., 2023). Clinical intervention should focus on stabilizing iron metabolism and blocking ferroptosis to improve the maternal pregnancy environment (Figure 4C). In endometriosis-associated infertility, iron overload in peritoneal fluid impairs mouse blastocyst formation through GPX4 downregulation, elevated lipid peroxidation, mitochondrial damage characterized by decreased ATP and increased ROS, mitochondrial membrane hyperpolarization, and HMOX1 (heme oxygenase 1) upregulation as a compensatory protective response, ultimately resulting in impaired blastocyst formation (Figure 4C). Maternal pathological conditions, through intrauterine microenvironment-mediated iron metabolism disorders, initiate a cascade amplification of fetal ferroptosis from the source. Clinical interventions, by targeting and stabilizing iron metabolism and blocking cascade amplification circuits, can effectively improve the intrauterine environment, reduce the risk of adverse pregnancy outcomes, and provide a precise theoretical basis for the prevention and treatment of perinatal diseases (Chang et al., 2023).

## 5 Advances in embryonic ferroptosis detection systems, model construction, and research methods

### 5.1 Construction and application of *in vivo* and *in vitro* model system of embryonic ferroptosis

**FIGURE 5:**
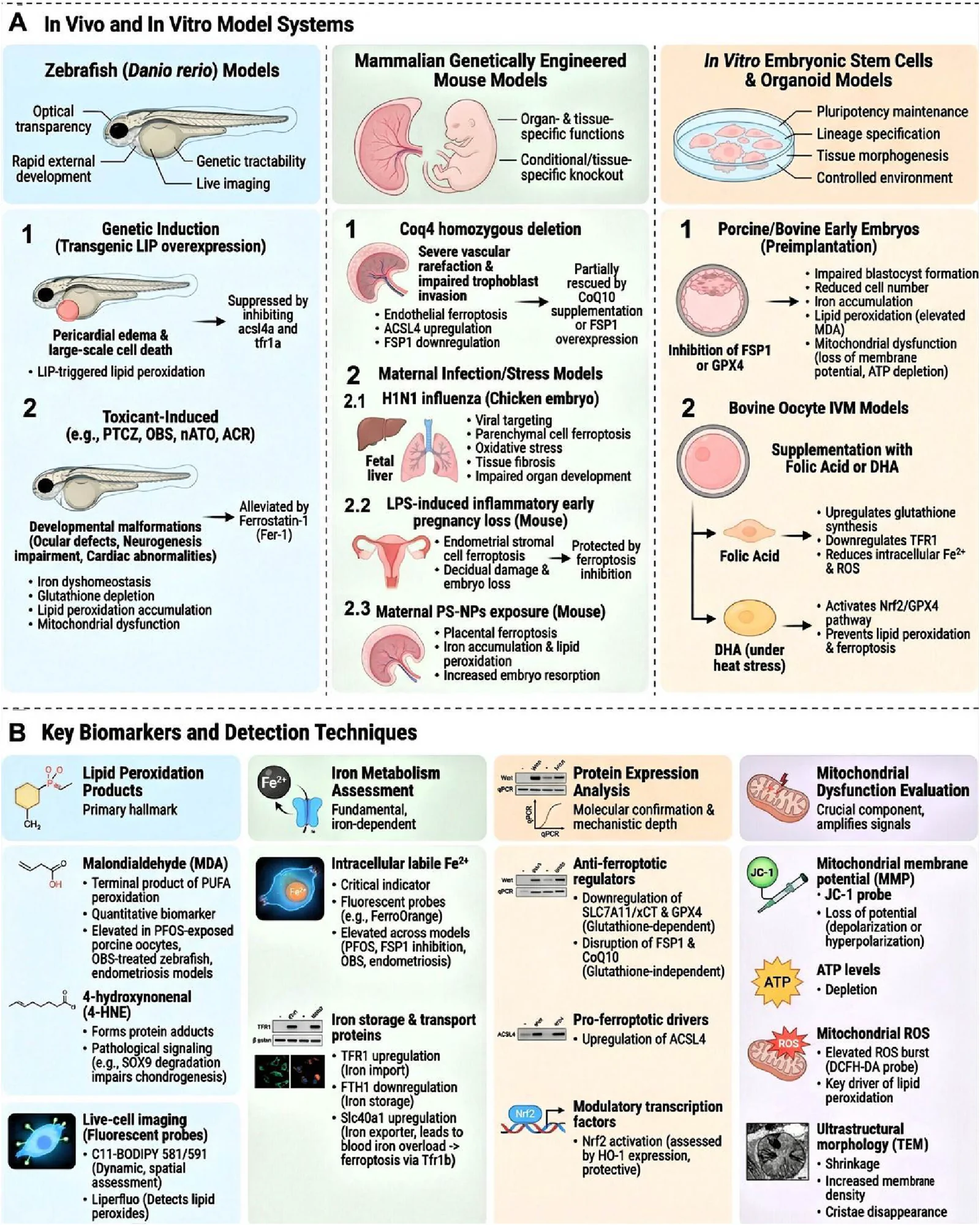
Typical model system and standardized detection strategy for ferroptosis research. **(A)** *In vivo* and *in vitro* model systems: *In vivo* models such as zebrafish (*Danio rerio*) and genetically engineered mice enable dynamic tracking of ferroptosis-related physiological and pathological phenotypes. Zebrafish models offer optical transparency, rapid external development, genetic tractability, and live imaging capabilities. Genetic induction through transgenic labile iron pool (LIP) overexpression triggers pericardial edema and large-scale cell death via LIP-triggered lipid peroxidation, which can be suppressed by inhibiting acsl4a and tfr1a. Toxicant-induced models (e.g., PTCZ, OBS, nATO, ACR) cause developmental malformations including ocular defects, neurogenesis impairment, and cardiac abnormalities, characterized by iron dyshomeostasis, glutathione depletion, lipid peroxidation accumulation, and mitochondrial dysfunction; these effects can be alleviated by Ferrostatin-1 (Fer-1). In mammalian genetically engineered mouse models, Coq4 homozygous deletion causes severe vascular rarefaction and impaired trophoblast invasion, endothelial ferroptosis, ACSL4 upregulation, and FSP1 downregulation, which can be partially rescued by CoQ10 supplementation or FSP1 overexpression. Maternal infection/stress models include H1N1 influenza in chicken embryos, which causes viral targeting, parenchymal cell ferroptosis, oxidative stress, tissue fibrosis, and impaired organ development in fetal liver; LPS-induced inflammatory early pregnancy loss in mice, which causes endometrial stromal cell ferroptosis, decidual damage, and embryo loss, can be protected by ferroptosis inhibition; and maternal PS-NPs exposure in mice, which causes placental ferroptosis, iron accumulation and lipid peroxidation, and increased embryo resorption. *In vitro* embryonic stem cell and organoid models provide a controllable and quantifiable experimental platform for the analysis of ferroptosis mechanisms and the evaluation of intervention strategies, including porcine/bovine early embryos (preimplantation), where inhibition of FSP1 or GPX4 impairs blastocyst formation, reduces cell number, causes iron accumulation, elevates lipid peroxidation (elevated MDA), and induces mitochondrial dysfunction characterized by loss of membrane potential and ATP depletion. Bovine oocyte *in vitro* maturation (IVM) models demonstrate that folic acid supplementation upregulates glutathione synthesis, downregulates TFRC, reduces intracellular Fe^2+^ and ROS; while DHA supplementation under heat stress activates the NRF2/GPX4 pathway, prevents lipid peroxidation and ferroptosis. **(B)** Core biomarkers and detection technologies: Lipid peroxidation level, iron metabolism homeostasis indicators, expression of key proteins in ferroptosis, and mitochondrial functional parameters together constitute the core biomarker spectrum for ferroptosis detection. Lipid peroxidation products include malondialdehyde (MDA), the terminal product of PUFA peroxidation and quantitative biomarker elevated in PFOS-exposed porcine oocytes, OBS-treated zebrafish, and endometriosis models; and 4-hydroxynonenal (4-HNE), which forms protein adducts and exerts pathological signaling (e.g., SOX9 degradation impairing chondrogenesis). Live-cell imaging using fluorescent probes such as C11-BODIPY 581/591 (for dynamic, spatial assessment) and Liperfluo (which detects lipid peroxides) enables real-time visualization. Iron metabolism assessment includes intracellular labile Fe^2+^ as a critical indicator detected by fluorescent probes (e.g., FerroOrange), elevated across models including PFOS, FSP1 inhibition, and endometriosis; iron storage and transport proteins such as TFRC upregulation (iron import), FTH1 downregulation (iron storage), and Slc40a1 upregulation (iron exporter, leading to blood iron overload and ferroptosis via Tfr1b). Protein expression analysis through molecular confirmation and mechanistic depth evaluation includes anti-ferroptotic regulators such as SLC7A11/xCT and GPX4 (glutathione-dependent) and disruption of FSP1 and CoQ10 (glutathione-independent), as well as pro-ferroptotic drivers such as upregulation of ACSL4. Modulatory transcription factors include NRF2 activation (assessed by HO-1 expression, protective). Mitochondrial dysfunction evaluation is a crucial component that amplifies signals, including mitochondrial membrane potential (MMP) assessed by JC-1 probe (loss of potential through depolarization or hyperpolarization), ATP levels (depletion), mitochondrial ROS (elevated ROS burst detected by DCFH-DA probe, key driver of lipid peroxidation), and ultrastructural morphology by transmission electron microscopy (TEM) revealing shrinkage, increased membrane density, and cristae disappearance. Combined with multi-omics and functional verification technologies, precise characterization of the process, intensity and regulatory mechanism of ferroptosis can be achieved.

Zebrafish models are transparent and easy to manipulate, making them ideal tools for studying embryonic ferroptosis: Labile iron pool (LIP) overexpression activates the ferroptosis pathway, while acyl-CoA synthetase long-chain family member 4 (ACSL4) and transferrin receptor (TFRC) knockdown inhibits ferroptosis and reverses developmental damage, directly verifying the regulatory role of ferroptosis in embryonic developmental impairment (Jia et al., 2023). In zebrafish models, genetic induction through transgenic LIP overexpression triggers pericardial edema and large-scale cell death via LIP-triggered lipid peroxidation, which can be suppressed by inhibiting acsl4a and tfr1a. Toxicant-induced models (e.g., PTCZ, OBS, nATO, ACR) cause developmental malformations including ocular defects, neurogenesis impairment, and cardiac abnormalities, characterized by iron dyshomeostasis, glutathione depletion, lipid peroxidation accumulation, and mitochondrial dysfunction; these effects can be alleviated by Ferrostatin-1 (Fer-1) (Figure 5A).

In mammals, ferritin light chain (FTL) knockdown in rats induces a preeclampsia phenotype, and the ferroptosis inhibitor Ferrostatin-1 significantly alleviates the lesions, revealing the core mediating role of ferroptosis in preeclampsia-related placental dysfunction. In mammalian genetically engineered mouse models, Coq4 homozygous deletion causes severe vascular rarefaction and impaired trophoblast invasion, endothelial ferroptosis, ACSL4 upregulation, and FSP1 downregulation, which can be partially rescued by CoQ10 supplementation or FSP1 overexpression. Maternal infection/stress models include H1N1 influenza in chicken embryos, which causes viral targeting, parenchymal cell ferroptosis, oxidative stress, tissue fibrosis, and impaired organ development in fetal liver; LPS-induced inflammatory early pregnancy loss in mice, which causes endometrial stromal cell ferroptosis, decidual damage, and embryo loss, can be protected by ferroptosis inhibition; and maternal PS-NPs exposure in mice, which causes placental ferroptosis, iron accumulation and lipid peroxidation, and increased embryo resorption (Figure 5A).

*In vitro* models such as embryonic stem cells and oocytes provide important tools for elucidating the mechanisms of ferroptosis under controlled conditions: intervening in key targets of iron metabolism and lipid peroxidation in *in vitro* culture systems allows for direct observation of the ferroptosis process and precise quantification of cellular oxidative damage and apoptosis levels (Figure 5A). *In vitro* embryonic stem cell and organoid models include porcine/bovine early embryos (preimplantation), where inhibition of FSP1 or GPX4 impairs blastocyst formation, reduces cell number, causes iron accumulation, elevates lipid peroxidation (elevated MDA), and induces mitochondrial dysfunction characterized by loss of membrane potential and ATP depletion. Bovine oocyte *in vitro* maturation (IVM) models demonstrate that folic acid supplementation upregulates glutathione synthesis, downregulates TFRC, reduces intracellular Fe^2+^ and ROS; while DHA supplementation under heat stress activates the NRF2/GPX4 pathway, prevents lipid peroxidation and ferroptosis (Figure 5A).

The core research value of the aforementioned *in vivo* and *in vitro* models lies in accurately verifying the key regulatory nodes of the ferroptosis cascade amplification loop: the zebrafish model directly verifies the core mechanism of “iron overload → lipid peroxidation initiation → cascade amplification → developmental damage” by targeting iron metabolism (TFRC regulation) and lipid peroxidation (ACSL4 knockdown); the FTL knockdown rat model reproduces placental dysfunction mediated by ferroptosis cascade amplification by simulating iron overload, and Ferrostatin-1 effectively reverses cascade amplification damage by blocking the lipid peroxidation chain reaction, providing direct experimental evidence for targeted intervention of ferroptosis cascade amplification; *in vitro* models such as embryonic stem cells and oocytes can accurately analyze the regulatory roles of each molecule in the cascade amplification loop under controllable conditions, laying an important foundation for the translation of mechanism research into clinical practice (Ducreux et al., 2024). See Tables 1–4 for detailed summaries of research models, biomarkers, anti-ferroptosis agents and intervention-strategy comparisons.

**TABLE 1:** Classification and characteristics of embryonic ferroptosis research models.

Model category	Specific models	Core advantages	Ferroptosis phenotypes	Intervention outcomes	Limitations
Aquatic *in vivo* model	Zebrafish (wild-type/LIP overexpression)	Transparent embryos, rapid development, facile genetic manipulation, live imaging	LIP overexpression → pericardial edema, widespread cell death; toxicant exposure → iron overload, GPX4 downregulation, developmental defects	Fer-1 and astaxanthin reverse malformations; ACSL4/TFRC knockdown suppresses lipid peroxidation	Large interspecies gap; cannot recapitulate mammalian placental development
Genetically modified mammalian model	Coq4-KO mice, FTL-knockdown rats, GPX4 global/tissue-specific KO mice	Mimic human placental and embryonic features; tissue-specific KO clarifies gene function	Coq4 KO: placental vascular rarefaction, endothelial ferroptosis, embryonic lethality; GPX4 KO: embryonic death at E7.5	CoQ10/FSP1 overexpression partially rescues defects; Fer-1 ameliorates preeclampsia phenotype	High breeding cost, long cycle; incompletely mirrors human embryonic redox regulation
Maternal stress/infection model	LPS-induced miscarriage mice, H1N1-infected chick embryos, PS-NPs-exposed pregnant mice	Mimic clinical maternal pathological insults affecting fetal development	LPS: endometrial stromal ferroptosis, decidual damage, embryo resorption; H1N1: fetal liver ferroptosis, growth retardation	Ferroptosis inhibitors significantly reduce embryo loss and tissue fibrosis	Exposure window difficult to match precisely with human critical developmental stages
*In vitro* embryonic cell/organoid model	Porcine/bovine preimplantation embryos, bovine oocyte IVM model, mouse/human ESCs	Controllable environment, convenient molecular intervention, low ethical risk	FSP1/GPX4 inhibition: decreased blastocyst rate, mitochondrial dysfunction; heat stress: oocyte ferroptosis, meiotic failure	Folic acid/DHA/NAC activate NRF2-GPX4 axis and suppress ferroptosis	Lack of maternal-fetal interface microenvironment; cannot reflect *in vivo* tissue remodeling

**TABLE 2:** Core biomarkers and detection technologies for embryonic ferroptosis.

Biomarker class	Indicators	Features upon ferroptosis activation	Detection methods	Research/Clinical value
Lipid peroxidation end products (core diagnostic)	MDA, 4-HNE	Terminal products of PUFA peroxidation; markedly elevated under toxicant exposure/endometriosis	Colorimetric assay, immunofluorescence, western blot	Quantitative assessment of ferroptosis cascade intensity; candidate perinatal screening biomarkers
Real-time lipid peroxide probes	C11-BODIPY, liperfluo	Fluorescence shift directly labels membrane lipid peroxides	Live-cell confocal, *in vivo* embryo imaging	Dynamic tracking of ferroptosis “trigger wave” propagation in embryonic tissues
Iron metabolism markers	Labile Fe^2+^, TFRC, FTH1, SLC40A1	Elevated Fe^2+^ initiates fenton reaction; TFRC upregulated, FTH1 downregulated	FerroOrange probe, qPCR, IHC	Determine whether iron dysregulation serves as the upstream driver
Anti-ferroptotic proteins	GPX4, SLC7A11, FSP1, CoQ10	Downregulated when dual antioxidant defense collapses	Western blot, immunofluorescence	Evaluate integrity of the two core embryonic antioxidant axes
Pro-ferroptotic proteins	ACSL4, TXNIP, NCOA4	ACSL4 catalyzes PUFA-PL synthesis; TXNIP/NCOA4 mediate ferritinophagy iron release	qPCR, immunostaining	Identify key drivers of lipid peroxidation and iron release
Mitochondrial function (cascade amplification)	MMP, ATP, mitochondrial ROS, ultrastructure	Membrane potential dysregulation, ATP depletion, ROS burst, mitochondrial shrinkage with cristae loss	JC-1 probe, ATP assay, TEM	Assess secondary mitochondrial damage induced by ferroptosis
Transcriptional regulators	NRF2/HO-1	NRF2 nuclear translocation and HO-1 upregulation as compensatory protective response	Immunofluorescence, qPCR	Judge activation level of endogenous antioxidant repair system

**TABLE 3:** Classification, mechanisms and applications of anti-ferroptosis agents.

Intervention type	Representative agents	Molecular targets	Embryo protective effects	Limitations & clinical constraints
Classical radical-trapping inhibitors	Ferrostatin-1, Liproxstatin-1	Directly scavenge lipid radicals; block lipid peroxidation chain reaction	Alleviate toxicant-induced zebrafish malformations; reverse blastocyst ferroptosis in endometriosis	May interfere with physiological ferroptosis-mediated embryonic tissue sculpting; long-term fetal safety unclear
CoQ10/FSP1 pathway-targeted agents	Coenzyme Q10 supplementation, FSP1 overexpression	Activate non-GSH compensatory antioxidant pathway; stabilize mitochondrial membrane	Rescue placental vascular defects in Coq4-KO mice; improve porcine embryo quality after FSP1 inhibition	Gene overexpression raises prenatal ethical barriers; poor placental transport efficiency of exogenous CoQ10
GSH-precursor nutritional antioxidants	NAC, Glycine	Replenish cysteine substrate; boost GSH synthesis; restore GPX4 activity	Inhibit PFOS-induced oocyte ferroptosis; improve blastocyst developmental potential	Limited efficacy against severe iron overload injury; requires combination with iron chelators
Natural multi-target antioxidants	Astaxanthin, Puerarin, Cacalol	Activate NRF2/Keap1/HO-1 axis; upregulate GPX4/FSP1; chelate excess iron	Astaxanthin mitigates TPhP neurotoxicity in zebrafish; puerarin improves ASD hippocampal neurogenesis	Low membrane permeability; high doses may disrupt embryonic redox homeostasis
Iron chelators	Deferasirox	Block fenton reaction at source; reduce labile Fe^2+^ pool	Reverse DBP-induced embryonic neuronal ferroptosis; break iron overload positive feedback	Excessive chelation causes maternal-fetal iron deficiency and fetal growth restriction
Cell-based therapeutics	hUC-MSCs	Paracrine upregulation of GPX4/FSP1; repair blood-testis barrier and placental endothelium	Reverse cisplatin-induced germ cell ferroptosis and infertility; reduce placental ferroptosis	Paracrine factor concentration difficult to control; insufficient clinical safety data
Gene editing tools	CRISPR/Cas9	Precisely correct pathogenic mutations in NARFL, GPX4, TFRCetc.	Restore antioxidant protein function in gene-deficient embryo models; block ferroptosis initiation	Strict ethical restrictions on human embryo editing; off-target mutation risk

**TABLE 4:** Comparison of three targeted intervention strategies for embryonic ferroptosis.

Strategy category	Core rationale	Key targets	Applicable scenarios	Translational Advantages & challenges
Combined nutritional antioxidant intervention (most translational)	Multi-node repair of dual antioxidant defense; block cascade amplification	NAC + Glycine (GSH precursors) + Astaxanthin (NRF2 agonist) + low-dose iron chelator	1. ART embryo culture medium optimization 2. Perinatal intervention for pollutant-exposed pregnancies 3. Adjuvant protection in mild endometriosis-related infertility	Advantages: High safety, low cost, no ethical concerns Challenges: Weak targeting; limited efficacy against severe genetic ferroptosis defects
Small-molecule ferroptosis inhibitor therapy	Directly interrupt lipid peroxidation chain reaction; suppress pathological ferroptosis burst	Fer-1/Liproxstatin-1, CoQ10, NRF2 small-molecule activators	1. Preclinical intervention in teratogen-exposed animal models 2. Short-term *in vitro* embryo protection in assisted reproduction	Advantages: Rapid onset, well-defined molecular targets Challenges: May off-target physiological tissue-remodeling ferroptosis; long-term fetal safety undetermined
Gene-cell precision therapy (long-term prospect)	Fundamentally correct upstream iron/lipid metabolism gene defects; permanently restore antioxidant balance	1. hUC-MSC paracrine protection 2. CRISPR repair of NARFL/GPX4 mutations	1. Inherited embryonic developmental defects (Coq4/NARFL deficiency) 2. Severe chemotherapeutic (cisplatin) reproductive toxicity	Advantages: Etiological treatment, high specificity Challenges: Strict human embryo ethics; immune rejection and gene off-target risks

### 5.2 Key biomarkers and precision detection technologies for ferroptosis

Lipid peroxidation is a core characteristic of ferroptosis, and malondialdehyde (MDA) and 4-hydroxynonenal (4-HNE) are key quantitative biomarkers for embryonic ferroptosis research (Zhang X. et al., 2023). MDA is the terminal product of PUFA peroxidation, serving as a quantitative biomarker elevated in PFOS-exposed porcine oocytes, OBS-treated zebrafish, and endometriosis models. 4-HNE forms protein adducts and exerts pathological signaling (e.g., SOX9 degradation impairing chondrogenesis). Live-cell imaging using fluorescent probes such as C11-BODIPY 581/591 (for dynamic, spatial assessment) and Liperfluo (which detects lipid peroxides) enables real-time visualization of ferroptosis progression (Figure 5B).

The ferroptosis inducer RAS-selective lethal 3 (RSL3) significantly increases MDA levels in porcine oocytes, while 4-HNE directly reflects the cascade amplification effect of lipid peroxidation. Curcumin supplementation can simultaneously inhibit the increase of both biomarkers through antioxidant action, thus blocking ferroptosis and alleviating damage. These biomarkers provide crucial evidence for the detection and mechanistic study of ferroptosis (Figure 5B). Iron metabolism assessment includes intracellular labile Fe^2+^ as a critical indicator detected by fluorescent probes (e.g., FerroOrange), which is elevated across models including PFOS, FSP1 inhibition, and endometriosis. Iron storage and transport proteins such as TFRC upregulation (iron import), FTH1 downregulation (iron storage), and Slc40a1 upregulation (iron exporter, leading to blood iron overload and ferroptosis via Tfr1b) provide complementary mechanistic insights. Protein expression analysis through molecular confirmation and mechanistic depth evaluation includes anti-ferroptotic regulators such as SLC7A11/xCT and GPX4 (glutathione-dependent) and disruption of FSP1 and CoQ10 (glutathione-independent), as well as pro-ferroptotic drivers such as upregulation of ACSL4. Modulatory transcription factors include NRF2 activation (assessed by HO-1 expression, protective). Mitochondrial dysfunction evaluation is a crucial component that amplifies signals, including mitochondrial membrane potential (MMP) assessed by JC-1 probe (loss of potential through depolarization or hyperpolarization), ATP levels (depletion), mitochondrial ROS (elevated ROS burst detected by DCFH-DA probe, key driver of lipid peroxidation), and ultrastructural morphology by transmission electron microscopy (TEM) revealing shrinkage, increased membrane density, and cristae disappearance (Figure 5B).

As end products of lipid peroxidation, MDA and 4-HNE directly quantify the activation intensity of the ferroptosis cascade amplification: the cascade amplification of LPO after iron overload-induced lipid peroxidation is directly manifested as a significant increase in the levels of both biomarkers, thus accurately reflecting the degree of pathological damage. RSL3 increases MDA levels by inhibiting GPX4 activity and disrupting antioxidant defenses, thereby accelerating the lipid peroxidation cascade. This demonstrates its core value in mechanism validation. Curcumin, on the other hand, prevents LPO generation and cascade amplification from the source by scavenging ROS and stabilizing dual antioxidant pathways, achieving downregulation of both and reversal of damage. This provides important theoretical support for targeted detection and nutritional intervention (Kurniawan et al., 2025).

## 6 Targeted intervention strategies for ferroptosis: gene and cell therapy combined with nutritional antioxidant regulation

### 6.1 Application prospects of ferroptosis inhibitors

**FIGURE 6:**
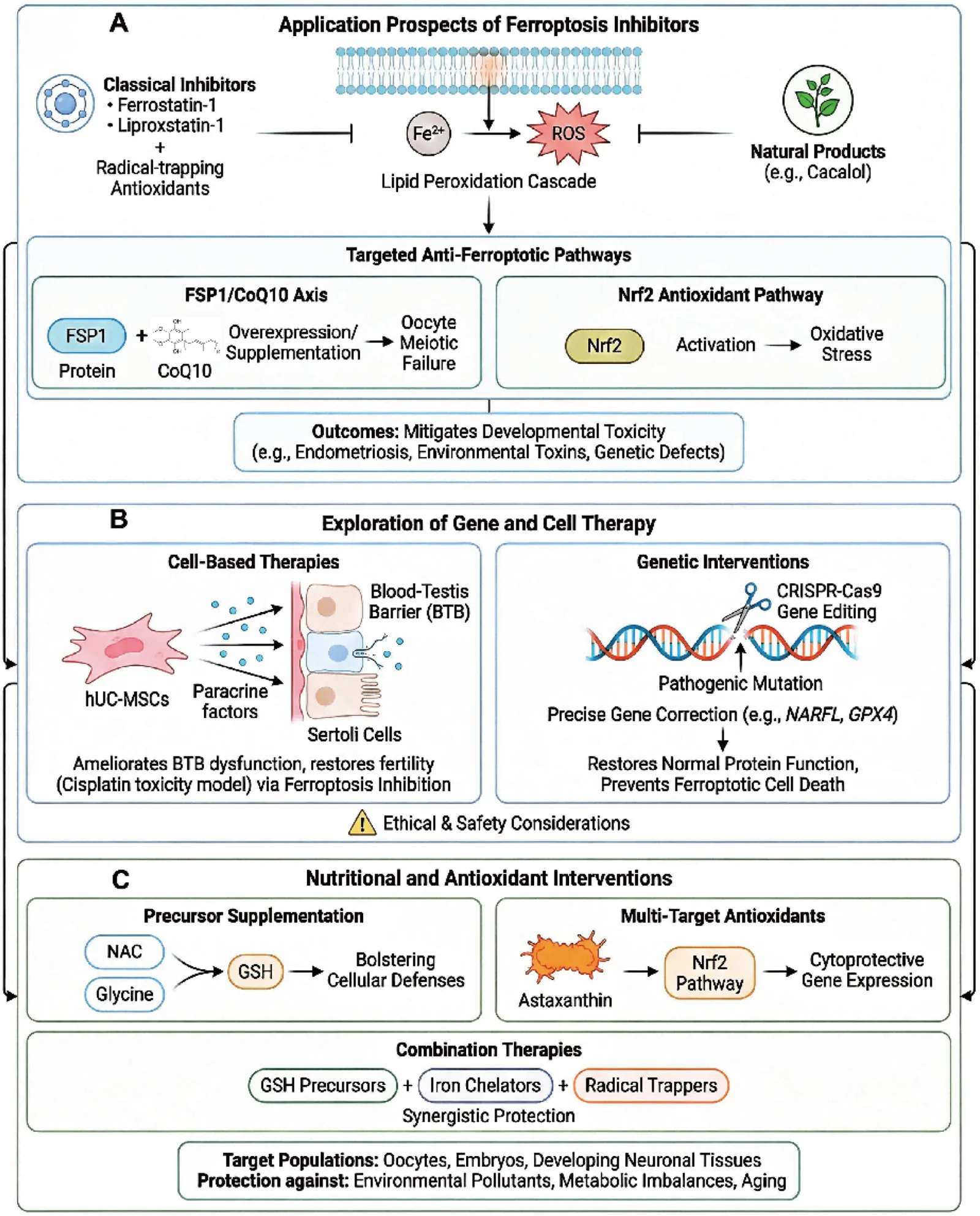
Targeted intervention strategies and application prospects for embryonic ferroptosis. **(A)** Application prospects of ferroptosis inhibitors: Classical inhibitors including Ferrostatin-1 and Liproxstatin-1 function as radical-trapping antioxidants that block the lipid peroxidation cascade by intercepting lipid free radicals and preventing ROS generation. Natural products (e.g., cacarol) provide alternative intervention candidates. Targeted anti-ferroptotic pathways include the FSP1/CoQ10 axis, where FSP1 protein overexpression or CoQ10 supplementation rescues oocyte meiotic failure, and the NRF2 antioxidant pathway, where NRF2 activation reduces oxidative stress. These interventions mitigate developmental toxicity outcomes including endometriosis, environmental toxin-induced damage, and genetic defects. **(B)** Exploration of gene and cell therapy: Cell-based therapies utilize human umbilical cord mesenchymal stem cells (hUC-MSCs) that release paracrine factors to ameliorate blood-testis barrier (BTB) dysfunction and restore fertility in cisplatin toxicity models via ferroptosis inhibition, acting on Sertoli cells to repair the BTB. Genetic interventions employ CRISPR-Cas9 gene editing to enable precise gene correction of pathogenic mutations (e.g., NARFL, GPX4), restoring normal protein function and preventing ferroptotic cell death. These approaches require careful ethical and safety considerations. **(C)** Nutritional and antioxidant intervention strategies: Precursor supplementation with N-acetylcysteine (NAC) and glycine bolsters cellular defenses by replenishing glutathione (GSH). Multi-target antioxidants such as astaxanthin activate the NRF2 pathway to induce cytoprotective gene expression. Combination therapies integrating GSH precursors, iron chelators, and radical trappers provide synergistic protection. These interventions target oocytes, embryos, and developing neuronal tissues, offering protection against environmental pollutants, metabolic imbalances, and aging-related damage.

Excessive ferroptosis activation is closely associated with various embryonic developmental toxicities, and targeted inhibition of ferroptosis has become one of the core strategies for embryo protection. Classical ferroptosis inhibitors (such as Ferrostatin-1 and Liproxstatin-1) can alleviate zebrafish embryonic developmental toxicity induced by environmental toxins (such as prothioconazole) and regulate erythropoiesis in rat embryos by directly capturing lipid free radicals and blocking lipid peroxidation, and can also regulate the process of erythropoiesis in rat embryos. Novel inhibitors (such as Ogremorphin) and natural products (such as cacarol) provide safe candidates for intervention during pregnancy due to their advantages of low toxicity and high selectivity. Pathway-targeted interventions have also shown clear effects: in a Coq4-deficient mouse model, supplementation with CoQ10 or overexpression of FSP1 can alleviate placental vascular development defects; activation of the Nrf2 endogenous antioxidant pathway can also reduce embryonic oxidative stress and ferroptosis damage (Figure 6A). Classical inhibitors including Ferrostatin-1 and Liproxstatin-1 function as radical-trapping antioxidants that block the lipid peroxidation cascade by intercepting lipid free radicals. The FSP1/CoQ10 axis can be targeted through FSP1 protein overexpression or CoQ10 supplementation to rescue oocyte meiotic failure. The NRF2 antioxidant pathway can be activated to reduce oxidative stress. These targeted anti-ferroptotic pathways mitigate developmental toxicity outcomes including endometriosis, environmental toxin-induced damage, and genetic defects (Figure 6A). These interventions provide feasible directions for the prevention and treatment of developmental defects related to environmental and genetic factors.

### 6.2 Exploring gene and cell-targeted therapies for ferroptosis

Ferroptosis is a core regulatory node in embryonic development, and gene and cell therapies offer new avenues for precise intervention in ferroptosis-related developmental disorders. Human umbilical cord mesenchymal stem cells (hUC-MSCs) can inhibit intracellular ferroptosis through paracrine effects, repair the structure and function of the blood-testis barrier (BTB), effectively reverse cisplatin-induced reproductive damage, and thus improve embryonic developmental potential. Clustered regularly interspaced short palindromic repeats/CRISPR-associated protein 9 (CRISPR/Cas9) technology can precisely edit iron metabolism-related genes: hepcidin knockout in zebrafish can induce ferroptosis and developmental delay, while neurodevelopmental defects caused by ATPase copper transporting alpha (ATP7A) deficiency can be significantly improved by ferroptosis inhibitors (Figure 6B). In cell-based therapies, hUC-MSCs release paracrine factors that ameliorate BTB dysfunction and restore fertility in cisplatin toxicity models via ferroptosis inhibition, acting on Sertoli cells to repair the blood-testis barrier. In genetic interventions, CRISPR-Cas9 gene editing enables precise gene correction of pathogenic mutations (e.g., NARFL, GPX4), restoring normal protein function and preventing ferroptotic cell death. These approaches require careful ethical and safety considerations (Figure 6B). Genes and cell therapies provide a potential clinical translational pathway for ferroptosis-mediated developmental abnormalities. The core mechanism of gene and cell therapy lies in precisely disrupting key nodes in the ferroptosis cascade amplification loop: hUC-MSCs directly block the LPO accumulation and lipid peroxidation chain reaction by paracrine upregulating GPX4 and FSP1 expression, inhibiting the cascade amplification downstream; CRISPR/Cas9-targeted editing of iron transport genes (such as TFRC and FTL) reduces the initiation of iron overload at the source, cutting off the core driving force of the pathological loop; ferroptosis inhibitors interrupt the positive feedback loop by clearing ROS and inhibiting lipid peroxidation. These strategies, by blocking the cascade amplification at multiple stages, provide an efficient and precise technical pathway for the clinical translation of embryonic developmental abnormalities (Mattar et al., 2024).

### 6.3 Nutritional and antioxidant intervention strategies for ferroptosis

Nutritional and antioxidant interventions are key safety strategies for blocking ferroptosis and protecting embryonic development. Glycine and N-acetylcysteine (NAC) can inhibit ferroptosis and promote oocyte maturation and embryonic development by enhancing antioxidant defense, inhibiting ROS accumulation and lipid peroxidation; in a zinc oxide (ZnO) nanoparticle neurotoxicity model, NAC can effectively alleviate oxidative stress and cell death. Natural antioxidants such as astaxanthin can inhibit the ferroptosis cascade at its source by activating the NRF2 pathway. Phosphatidylethanolamine metabolism is closely related to ferroptosis, and its nutritional regulation has potential neuroprotective value (Figure 6C). Precursor supplementation with NAC and glycine bolsters cellular defenses by replenishing GSH. Multi-target antioxidants such as astaxanthin activate the NRF2 pathway to induce cytoprotective gene expression. Combination therapies integrating GSH precursors, iron chelators, and radical trappers provide synergistic protection. These interventions target oocytes, embryos, and developing neuronal tissues, offering protection against environmental pollutants, metabolic imbalances, and aging-related damage (Figure 6C). These interventions, due to their high safety and accessibility, have significant translational implications for the control of environmental developmental toxicity. The core role of nutritional and antioxidant intervention lies in systematically repairing the defense barrier of the ferroptosis cascade amplification circuit: glycine reduces the generation of lipid peroxidation substrates at the source by maintaining cell membrane integrity and inhibiting the synthesis of polyunsaturated fatty acids; NAC, as a direct reducing agent, rapidly clears ROS and replenishes GSH reserves, restoring the function of the classical GPX4-GSH antioxidant pathway and blocking the lipid peroxidation chain reaction; astaxanthin activates the NRF2 transcription factor, upregulates the expression of core antioxidant enzymes such as GPX4 and FSP1, constructs a systemic defense network, and curbs the initiation of the iron overload-mediated cascade amplification at the source. These strategies, by blocking the cascade circuit from multiple dimensions—substrate, pathway, and transcription—provide a safe and efficient practical approach for perinatal nutritional management and environmental toxicity control in cases of abnormal embryonic development (Gao Y. et al., 2026).

## 7 Challenges and future prospects in the study of ferroptosis in embryonic development

### 7.1 The depth and complexity of research on the regulatory mechanisms of ferroptosis

**FIGURE 7:**
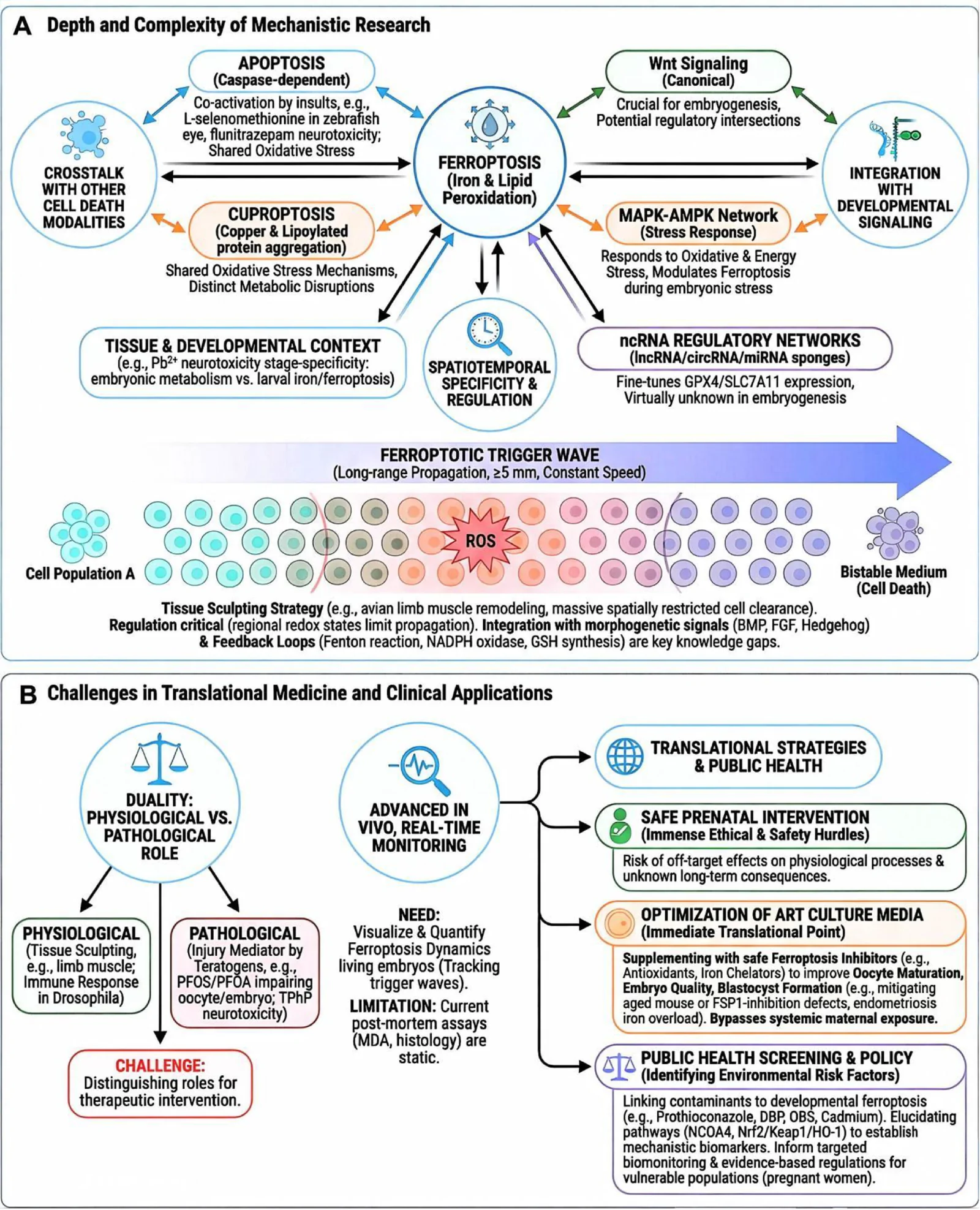
Challenges and future prospects in the study of ferroptosis in embryonic development. **(A)** Depth and complexity of mechanistic research: Ferroptosis (iron and lipid peroxidation) exhibits extensive crosstalk with other cell death modalities. Apoptosis (caspase-dependent) shows co-activation by insults such as L-selenomethionine in zebrafish eye and flunitrazepam neurotoxicity, with shared oxidative stress as a common mechanistic node. Cuproptosis (copper and lipoylated protein aggregation) shares oxidative stress mechanisms but exhibits distinct metabolic disruptions. The MAPK-AMPK network responds to oxidative and energy stress, modulating ferroptosis during embryonic stress. Non-coding RNA (ncRNA) regulatory networks including lncRNA, circRNA, and miRNA sponges fine-tune GPX4/SLC7A11 expression, though their roles in embryogenesis remain virtually unknown. Wnt signaling, crucial for embryogenesis, presents potential regulatory intersections with ferroptosis pathways. Spatiotemporal specificity and regulation are exemplified by the ferroptotic trigger wave—long-range propagation at constant speed (≥5 mm) across cell populations from a bistable medium to cell death, representing a tissue sculpting strategy (e.g., avian limb muscle remodeling, massive spatially restricted cell clearance). Regulation of these trigger waves is critical, with regional redox states limiting propagation, and integration with morphogenetic signals (BMP, FGF, Hedgehog) and feedback loops (Fenton reaction, NADPH oxidase, GSH synthesis) representing key knowledge gaps. Tissue and developmental context (e.g., Pb^2+^ neurotoxicity stage-specificity; embryonic metabolism vs. larval iron/ferroptosis) further modulates ferroptosis regulation. **(B)** Challenges in translational medicine and clinical applications: The duality of physiological versus pathological roles of ferroptosis presents a core challenge—distinguishing roles for therapeutic intervention requires careful consideration of tissue sculpting, trigger waves, and immune response in *Drosophila* as physiological functions, versus injury mediation by teratogens (e.g., PFOS/PFOA impairing oocyte/embryo, TPhP neurotoxicity) as pathological outcomes. Advanced *in vivo*, real-time monitoring is needed to visualize and quantify ferroptosis dynamics in living embryos, particularly for tracking trigger waves; current limitation is that post-mortem assays (MDA, histology) are static. Translational strategies and public health applications include safe prenatal intervention, which faces immense ethical and safety hurdles due to risks of off-target effects on physiological processes and unknown long-term consequences. Optimization of assisted reproductive technology (ART) culture media represents an immediate translational point: supplementing with safe ferroptosis inhibitors (e.g., antioxidants, iron chelators) to improve oocyte maturation, embryo quality, and blastocyst formation (e.g., mitigating aged mouse or FSP1-inhibition defects, endometriosis iron overload), bypassing systemic maternal exposure. Public health screening and policy should focus on identifying environmental risk factors, linking contaminants to developmental ferroptosis pathways (NCOA4, NRF2/Keap1/HO-1), elucidating pathways to establish mechanistic biomarkers, and informing targeted biomonitoring and evidence-based regulations for vulnerable populations (pregnant women).

Ferroptosis in embryonic development exhibits high spatiotemporal specificity and pathway cross-regulation: iron homeostasis, lipid peroxidation cascades, and antioxidant defense systems are differentially regulated at different developmental stages and in different tissues and organs. Furthermore, multiple factors, including maternal nutrition, environmental exposure, and epigenetic modifications, further couple and intervene in this process. Current research is largely limited to *in vitro* cell models and model organisms; the *in-situ* regulatory network of ferroptosis in human embryonic development and its interactive regulatory mechanisms with other cell death modes such as apoptosis or autophagy remain incompletely elucidated (Figure 7A). From the perspective of mechanistic depth and complexity, ferroptosis exhibits extensive crosstalk with other cell death modalities: apoptosis (caspase-dependent) shows co-activation by insults such as L-selenomethionine in zebrafish eye and flunitrazepam neurotoxicity, with shared oxidative stress as a common mechanistic node. Cuproptosis (copper and lipoylated protein aggregation) shares oxidative stress mechanisms but exhibits distinct metabolic disruptions. The MAPK-AMPK network responds to oxidative and energy stress, modulating ferroptosis during embryonic stress. Non-coding RNA (ncRNA) regulatory networks including lncRNA, circRNA, and miRNA sponges fine-tune GPX4/SLC7A11 expression, though their roles in embryogenesis remain virtually unknown. Wnt signaling, crucial for embryogenesis, presents potential regulatory intersections with ferroptosis pathways. Spatiotemporal specificity and regulation are exemplified by the ferroptotic trigger wave—long-range propagation at constant speed (≥5 mm) across cell populations from a bistable medium to cell death, representing a tissue sculpting strategy (e.g., avian limb muscle remodeling, massive spatially restricted cell clearance). Regulation of these trigger waves is critical, with regional redox states limiting propagation, and integration with morphogenetic signals (BMP, FGF, Hedgehog) and feedback loops (Fenton reaction, NADPH oxidase, GSH synthesis) representing key knowledge gaps (Figure 7A).

From the perspective of ferroptosis cascade amplification, the core research challenge lies in elucidating the developmental stage-specific cascade regulatory network: differences in iron metabolic requirements and lipid peroxidation levels at different embryonic developmental stages lead to significant tissue specificity in the initiation threshold and regulatory pathways of cascade amplification; simultaneously, the coupling of multiple factors and the cross-regulation of cell death modes further increase the complexity of mechanism elucidation. Future research should focus on elucidating the *in-situ* regulatory network of human embryos and developing precise intervention strategies targeting key nodes of cascade amplification to lay a theoretical foundation for clinical translation (Ma et al., 2026).

### 7.2 Challenges in translational medicine and clinical applications of ferroptosis

Based on the molecular mechanisms of ferroptosis, screening environmental teratogenic risk factors and developing protective strategies are of significant importance in preventive medicine. Various environmental pollutants (such as nano-zinc oxide and pesticides) can interfere with embryonic development by inducing ferroptosis, and ferroptosis-related molecular markers can serve as sensitive biomarkers for evaluating the developmental toxicity of chemicals (Figure 7B). From the perspective of translational medicine and clinical applications, the duality of physiological versus pathological roles of ferroptosis presents a core challenge: distinguishing roles for therapeutic intervention requires careful consideration of tissue sculpting, trigger waves, and immune response in *Drosophila* as physiological functions, versus injury mediation by teratogens (e.g., PFOS/PFOA impairing oocyte/embryo, TPhP neurotoxicity) as pathological outcomes. Advanced *in vivo*, real-time monitoring is needed to visualize and quantify ferroptosis dynamics in living embryos, particularly for tracking trigger waves; current limitation is that post-mortem assays (MDA, histology) are static. Translational strategies and public health applications include safe prenatal intervention, which faces immense ethical and safety hurdles due to risks of off-target effects on physiological processes and unknown long-term consequences. Optimization of assisted reproductive technology (ART) culture media represents an immediate translational point: supplementing with safe ferroptosis inhibitors (e.g., antioxidants, iron chelators) to improve oocyte maturation, embryo quality, and blastocyst formation (e.g., mitigating aged mouse or FSP1-inhibition defects, endometriosis iron overload), bypassing systemic maternal exposure. Public health screening and policy should focus on identifying environmental risk factors, linking contaminants to developmental ferroptosis pathways (NCOA4, NRF2/Keap1/HO-1), elucidating pathways to establish mechanistic biomarkers, and informing targeted biomonitoring and evidence-based regulations for vulnerable populations (pregnant women) (Figure 7B). Future research should utilize model organisms and high-throughput screening platforms to establish a chemical safety limit evaluation system centered on key ferroptosis molecules such as ACSL4, providing a scientific basis for revising chemical safety limits and protecting maternal health (Wang and Xie, 2025).

## 8 Discussion

Ferroptosis acts as a double-edged sword during embryonic development, and its cascade amplification effect is a pivotal driver shifting physiological regulation toward pathological damage. This work systematically sorts out the three core regulatory axes governing embryonic ferroptosis, namely the GPX4-GSH classical antioxidant pathway, the FSP1-CoQ10 compensatory pathway, and the iron-lipid peroxidation network. The synergistic operation of the dual antioxidant defense systems maintains redox homeostasis under normal conditions, which is essential for cell fate determination and tissue remodeling in early embryos. Once iron metabolism is disturbed, a closed positive feedback loop will be triggered, rapidly amplifying local oxidative damage into systemic developmental lesions.

This cascade amplification mechanism well explains why multiple exogenous hazards and maternal adverse states can easily induce embryonic malformations, growth restriction and even embryo loss. Environmental pollutants, clinical drugs and maternal complications all disrupt the upstream links of ferroptosis regulation, break the balance of the dual antioxidant pathways, and ultimately activate the irreversible lipid peroxidation chain reaction. It also accounts for the distinct sensitivity to ferroptosis among embryonic cells at different developmental stages and across species, which is closely tied to the expression level of core protective molecules and the completeness of complementary defense networks.

However, several critical issues warrant careful consideration. The cascade amplification model, while intuitively appealing, may oversimplify the complexity of ferroptosis regulation. As noted in the preceding sections, ferroptosis can be directly induced by GPX4 inhibition, cysteine deprivation, or ACSL4 upregulation without requiring prior iron overload as an initiating factor. This suggests that the “iron overload → lipid peroxidation → cascade amplification” paradigm is not the exclusive trigger mechanism, and alternative initiation pathways deserve equal attention in future research. The embryonic lethality observed in GPX4 systemic knockout mice, while commonly attributed to uncontrolled ferroptosis, may not be exclusively caused by this cell death modality. Concurrent activation of apoptosis, necrosis, or other cell death pathways may contribute to the phenotype, and the specific contribution of ferroptosis to embryonic lethality remains a matter of active debate. Disentangling the relative contributions of different cell death mechanisms in genetic knockout models represents a significant technical and conceptual challenge.

The current body of evidence relies heavily on *in vitro* cell culture systems and animal models (zebrafish, mouse, pig), and direct extrapolation to human embryonic development requires substantial caution. Interspecies differences in redox regulation, iron metabolism, and ferroptosis sensitivity may be considerable. For instance, zebrafish hepcidin gene knockout primarily results in iron overload rather than pronounced developmental delay, a phenotypic outcome that differs substantially from mammalian systems. Similarly, the comparative analysis of naïve mouse embryonic stem cells versus primed human embryonic stem cells, while informative, does not fully capture the complexity of *in vivo* human embryonic development. The development of human embryo models, such as extended *in vitro* culture of human blastocysts or blastoid organoids, may help bridge this gap, though ethical constraints limit their widespread application.

The clinical translation of ferroptosis inhibitors for embryonic protection faces significant hurdles. While ferrostatin-1, liproxstatin-1, and other radical-trapping antioxidants show promise in preclinical models, their specificity for ferroptosis versus other redox-sensitive processes is not absolute. Many of these compounds exhibit broad antioxidant activities that may interfere with normal physiological processes, including the trigger wave-mediated tissue sculpting essential for proper embryonic morphogenesis. The risk of off-target effects on physiological ferroptosis, particularly during critical windows of development, raises substantial safety concerns that have not been adequately addressed in current literature. Moreover, the long-term consequences of *in utero* ferroptosis inhibition remain virtually unknown, necessitating rigorous follow-up studies in appropriate model systems.

The interaction between ferroptosis and other programmed cell death modalities—apoptosis, autophagy (particularly ferritinophagy), cuproptosis, and necrosis—represents a critical area that remains incompletely understood. These pathways are not mutually exclusive but form an interconnected network with shared regulatory nodes and potential compensatory relationships. For example, pharmacological inhibition of ferroptosis may redirect cells toward apoptosis or other alternative death mechanisms, potentially masking rather than resolving the underlying developmental pathology. A comprehensive understanding of the cell death network during embryonic development, rather than a isolated focus on ferroptosis, is essential for developing effective therapeutic strategies.

In addition, the research models, characteristic biomarkers and diverse intervention strategies summarized in this paper build a complete technical and theoretical system for monitoring and intervening embryonic ferroptosis. Targeted regulation of key nodes in the cascade loop, including iron transport proteins, core antioxidant molecules and upstream signaling pathways, provides feasible ideas for blocking developmental toxicity caused by ferroptosis.

Nevertheless, multiple unresolved issues still remain in this field. The spatiotemporal specificity of ferroptosis regulation in human embryos, as well as the crosstalk between ferroptosis and other types of programmed cell death, have not been fully clarified. The current research mainly relies on model animals and *in vitro* cell systems, and there is still a large gap between existing findings and clinical practical application. Future studies need to further explore stage-specific regulatory characteristics of embryonic ferroptosis, optimize detection means and intervention regimens, and promote the translation of basic findings into clinical prevention and treatment of birth defects and pregnancy complications.

## 9 Conclusion

Ferroptosis is a precisely regulated form of cell death during embryonic development, possessing a dual nature: it is both a key executor of physiological morphological shaping and an important mediator of pathological damage. Research in this field has deepened our understanding of developmental biology, advanced research on embryotoxic mechanisms, and provided new directions for the prevention and treatment of developmental disorders. The ferroptosis cascade amplification effect is a core hub connecting iron metabolism disorders, oxidative stress, and abnormal embryonic development. Elucidating its mechanism provides a new theoretical framework and practical pathway for embryotoxicity control and perinatal intervention, possessing significant basic research and clinical translational value.

Moving forward, several priorities should guide future research. First, the development of advanced *in vivo* real-time monitoring technologies is urgently needed to visualize and quantify ferroptosis dynamics in living embryos, particularly for tracking trigger waves and distinguishing physiological from pathological ferroptosis in real time. Current post-mortem assays (MDA, histology) are static and cannot capture the spatiotemporal dynamics of ferroptosis propagation. Second, the establishment of human-specific model systems, including extended *in vitro* culture of human blastocysts and blastoid organoids, is essential to validate findings from animal models and bridge the gap between basic research and clinical application. Third, the optimization of intervention strategies must prioritize specificity and safety, particularly for prenatal applications. The potential off-target effects of broad-spectrum antioxidants on physiological ferroptosis-mediated tissue sculpting necessitate the development of stage-specific and tissue-specific modulators. Fourth, comprehensive regulatory frameworks and evidence-based policies should be established to identify environmental risk factors, link contaminants to developmental ferroptosis pathways, and inform targeted biomonitoring for vulnerable populations, particularly pregnant women. Addressing these challenges will accelerate the translation of ferroptosis research into effective strategies for preventing birth defects and improving pregnancy outcomes.

## Glossary

4-HNE: 4-hydroxynonenal
ACO1: Aconitase 1
ACR: Acrolein
ACSL4: Acyl-CoA Synthetase Long-Chain Family Member 4
ACSL4/TFRC: ACSL4 + Transferrin Receptor
acsl4a: acyl-CoA synthetase long-chain family member 4a
AIFM2: Apoptosis Inducing Factor Mitochondrial Associated 2
AMPK: AMP-Activated Protein Kinase
ART: Assisted Reproductive Technology
ASD: Autism Spectrum Disorder
ATP: Adenosine Triphosphate
ATP7A: ATPase Copper Transporting Alpha
CoA: Coenzyme A
CoQ10: Coenzyme Q10
**CoQ10H**_**2**_: Ubiquinol
Coq4: Coenzyme Q4 Biosynthesis Protein
CRISPR: Clustered Regularly Interspaced Short Palindromic Repeats
DBP: Dibutyl Phthalate
DCFH-DA: 2′,7′-Dichlorodihydrofluorescein Diacetate
DHA: Docosahexaenoic Acid
TFRC: Transferrin Receptor
TPhP: Triphenyl Phosphate
TXNIP: Thioredoxin-Interacting Protein
TXNIP/NCOA4: TXNIP-NCOA4 Ferritinophagy Axis
Wnt: Wingless/Integrated Signaling Pathway
xCT: Cystine Transporter SLC7A11
GPX4/SLC7A11: GPX4/Cystine Transporter SLC7A11
GRSF1: G-Rich RNA Binding Factor 1
GSH: Glutathione
H1N1: Influenza A Virus Subtype H1N1
HMOX1: Heme Oxygenase 1
HO-1: Heme Oxygenase 1
hUC-MSC: Human Umbilical Cord Mesenchymal Stem Cell
hUC-MSCs: Human Umbilical Cord Mesenchymal Stem Cells
ICM: Inner Cell Mass
IHC: Immunohistochemistry
IRP1: Iron Regulatory Protein 1
IRP2: Iron Regulatory Protein 2
IVM: *In Vitro* Maturation
Liperfluo: Liperfluo Lipid Peroxide Probe
Liproxstatin-1: Liproxstatin-1
lncRNA: Long Non-Coding RNA
LPCAT3: Lysophosphatidylcholine Acyltransferase 3
LPO: Lipid Peroxide
LPS: Lipopolysaccharide
ltf: Lactoferrin
MAPK-AMPK: Mitogen-Activated Protein Kinase - AMPK
MDA: Malondialdehyde
mESCs: Mouse Embryonic Stem Cells
mGPX4: Mitochondrial Glutathione Peroxidase 4
MMP: Mitochondrial Membrane Potential
MSC: Mesenchymal Stem Cell
NAC: N-Acetylcysteine
NAD(P)H: Nicotinamide Adenine Dinucleotide (Phosphate), Reduced Form
NADPH: Reduced Nicotinamide Adenine Dinucleotide Phosphate
NARFL: Nuclear Prelamin A Recognition Factor-Like
nATO: Sodium Antimonate
ncRNA: Non-Coding RNA
NCOA4: Nuclear Receptor Coactivator 4
NHLRC2: NHL Repeat Containing 2
ROS: Reactive Oxygen Species
RSL3: RAS-Selective Lethal 3
SLC3A2: Solute Carrier Family 3 Member 2
SLC40A1: Solute Carrier Family 40 Member 1
SLC7A11: Solute Carrier Family 7 Member 11
BBB: Blood-Brain Barrier
BCL-2: B-cell Lymphoma 2
bME: β-Mercaptoethanol
BMEC: Brain Microvascular Endothelial Cell
BMP: Bone Morphogenetic Protein
BTB: Blood-Testis Barrier
C11-BODIPY: C11-Boron-Dipyrromethene
Cas9: CRISPR-Associated Protein 9
CDDP: Cisplatin
cGPX4: Cytosolic Glutathione Peroxidase 4
CIA: Cytosolic Iron-Sulfur Cluster Assembly
CIAPIN1: Cytokine-Induced Apoptosis Inhibitor 1
circRNA: Circular RNA
Fer-1: Ferrostatin-1
FerroOrange: FerroOrange Fluorescent Probe
SSMD: Somatic Cell Mitochondrial Dysfunction
**System Xc** ^**-**^: Cystine-Glutamate Antiporter System Xc^-^
FGF: Fibroblast Growth Factor
FSP1: Ferroptosis Suppressor Protein 1
FSP1/GPX4: FSP1 + Glutathione Peroxidase 4
FTH: Ferritin Heavy Chain
fth: ferritin heavy chain
FTH1: Ferritin Heavy Chain 1
fth1b: ferritin heavy chain 1b
fthl: ferritin heavy chain-like
FTL: Ferritin Light Chain
GON4: Germ Cell-Less 4
GPX4: Glutathione Peroxidase 4
GPX4-GSH: GPX4-Glutathione Pathway
GPX4/FSP1: GPX4 + FSP1 Dual Antioxidant Axis
IRP2: Iron Regulatory Protein 2
IVM: In Vitro Maturation
JC-1: JC-1 Mitochondrial Membrane Potential Probe
Keap1: Kelch-Like ECH-Associated Protein 1
KO: Knockout
lgp4a: glutathione peroxidase 4a
LIP: Labile Iron Pool
NRF2: Nuclear Factor Erythroid 2-Related Factor 2
NRF2-GPX4: NRF2-GPX4 Antioxidant Axis
NRF2/GPX4: NRF2-GPX4
NSCs: Neural Stem Cells
OBS: Octabromodiphenyl Ether
OXPHOS: Oxidative Phosphorylation
p53: Tumor Protein p53
**Pb** ^**2+**^: Lead Ion
PCBP1: Poly(rC)-Binding Protein 1
PCBP2: Poly(rC)-Binding Protein 2
PFC: Prefrontal Cortex
PFOS: Perfluorooctane Sulfonate
PFOS/PFOA: PFOS + Perfluorooctanoic Acid
PLOHs: Phospholipid Hydroxides
PLOOHs: Phospholipid Hydroperoxides
PS-NPs: Polystyrene Nanoparticles
PTCZ: Prothioconazole
PUFA: Polyunsaturated Fatty Acid
PUFA-PL: PUFA-Containing Phospholipid
PUFAs: Polyunsaturated Fatty Acids
ZnO: Zinc Oxide Nanoparticles
RAS: Rat Sarcoma Viral Oncogene Homolog
slc7a11: solute carrier family 7 member 11
TEM: Transmission Electron Microscopy
TF: Transferrin
tfba: transferrin a
tfr1b: transferrin receptor 1b
SLC7A11/SLC3A2: System Xc^−^ Cystine Transporter Heterodimer
SOX9: SRY-Box Transcription Factor 9
